# Analysis of Cyp51 protein sequences shows 4 major Cyp51 gene family groups across fungi

**DOI:** 10.1093/g3journal/jkac249

**Published:** 2022-09-21

**Authors:** Brandi N Celia-Sanchez, Brandon Mangum, Marin Brewer, Michelle Momany

**Affiliations:** Department of Plant Biology, University of Georgia, Athens, GA 30606, USA; Department of Plant Biology, University of Georgia, Athens, GA 30606, USA; Department of Plant Pathology, University of Georgia, Athens, GA 30606, USA; Department of Plant Biology, University of Georgia, Athens, GA 30606, USA

**Keywords:** Cyp51, azole resistance, Cyp51A, Cyp51B, Cyp51C

## Abstract

Azole drugs target fungal sterol biosynthesis and are used to treat millions of human fungal infections each year. Resistance to azole drugs has emerged in multiple fungal pathogens including *Candida albicans*, *Cryptococcus neoformans*, *Histoplasma capsulatum*, and *Aspergillus fumigatus*. The most well-studied resistance mechanism in *A. fumigatus* arises from missense mutations in the coding sequence combined with a tandem repeat in the promoter of *cyp51A*, which encodes a cytochrome P450 enzyme in the fungal sterol biosynthesis pathway. Filamentous members of Ascomycota such as *A. fumigatus* have either 1 or 2 of 3 Cyp51 paralogs (Cyp51A, Cyp51B, and Cyp51C). Most previous research in *A. fumigatus* has focused on Cyp51A due to its role in azole resistance. We used the *A. fumigatus* Cyp51A protein sequence as the query in database searches to identify Cyp51 proteins across fungi. We found 435 Cyp51 proteins in 295 species spanning from early-diverging fungi (Blastocladiomycota, Chytridiomycota, Zoopagomycota, and Mucormycota) to late-diverging fungi (Ascomycota and Basidiomycota). We found these sequences formed 4 major Cyp51 groups: Cyp51, Cyp51A, Cyp51B, and Cyp51C. Surprisingly, we found all filamentous Ascomycota had a Cyp51B paralog, while only 50% had a Cyp51A paralog. We created maximum likelihood trees to investigate the evolution of Cyp51 in fungi. Our results suggest Cyp51 is present in all fungi with 3 paralogs emerging in Pezizomycotina, including Cyp51C which appears to have diverged from the progenitor of the Cyp51A and Cyp51B groups.

## Introduction

Fungal pathogens caused over 9 million diagnosed infections in 2017 in the United States, but the true fungal burden is hard to estimate since many cases are likely undiagnosed (Vallabhaneni *et al.* 2016; Benedict *et al.* 2019). The infections caused by fungal pathogens include severe chronic conditions, complex chronic respiratory conditions, recurrent infections, and many life-threatening invasive diseases (Perlin *et al.* 2017). Invasive fungal infections generally occur in individuals with suppressed or compromised immune systems (Brown *et al.* 2012). These infections have a high mortality rate if not treated early with appropriate antifungal drugs (Brown *et al.* 2012). Major drugs used to treat invasive fungal infections are echinocandins, polyenes, flucytosine, and azole drugs (Pound *et al.* 2011). Azoles, which target synthesis of the fungal-specific membrane component ergosterol, are among the most highly used antifungal drugs.

**Fig. 1.**
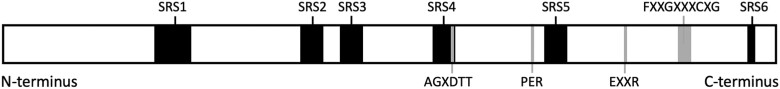
Typical organization of Cyp51 domains. Cyp51 proteins contain 6 substrate recognition sites (SRS1–6), an oxygen-binding motif (AGXDTT), PER and EXXR motifs, and a conserved heme-binding motif (FXXGXXXCXG). Black boxes represent SRS domains. Gray shading represents other motifs. Diagram is based on *A. fumigatus* Cyp51A (XP_752137.1) and is shown to scale.

Cyp51 proteins, also known as Erg11 in Ascomycota yeast in the Saccharomycotina and Taphrinomycotina subphyla, are in all biological kingdoms and are highly conserved (Lepesheva and Waterman 2011). Cyp51 proteins have 6 substrate recognition sites (SRS), an oxygen-binding motif (AGXDTT), PER, and EXXR motifs that create an E–R–R triad within the heme pocket, and a conserved heme-binding motif (FXXGXXXCXG) (Fig. 1) (Lepesheva and Waterman 2007; Córdova *et al.* 2017). Azole drugs competitively bind to sterol 14 alpha-demethylase (Cyp51, Erg11), a cytochrome P450 in the ergosterol biosynthesis pathway in fungi. Azole drugs consist of a heterocyclic ring with either 2 (imidazoles) or 3 (triazoles) nitrogens and a sidechain. The side chain of azoles interacts with the Cyp51 polypeptide while the nitrogen in the azole heterocyclic ring interacts directly with the sixth ligand of the heme ferric ion, a cofactor of the Cyp51 protein (Jefcoate *et al.* 1969). Cytochrome P450 proteins conduct a 3-step reaction within the sterol biosynthesis pathway leading to the production of cholesterol in animals, sitosterol in plants, and ergosterol in fungi (Schaller 2003; Dufourc 2008). Sterols are integrated into the cell membrane where they aid in membrane fluidity and permeability (Dufourc 2008). The binding of azoles to Cyp51 depletes intracellular ergosterol and causes accumulation of methylated sterols and toxic intermediate sterols within the fungal cell membrane causing arrested growth and cell membrane stress (Cowen and Steinbach 2008).

Many fungi have acquired mutations in *cyp51* that alter the ability of azoles to bind and inhibit Cyp51 (Denning and Perlin 2011; Leroux and Walker 2011; Flowers *et al.* 2015; Kano *et al.* 2017). Many missense mutations that have been shown to decrease sensitivity to azoles as determined by minimum inhibitory concentration in the human fungal pathogens *Candida albicans*, *Cryptococcus neoformans, Histoplasma capsulatum*, and *A. fumigatus* (Rodero *et al.* 2003; Wheat *et al.* 2006; Lockhart *et al.* 2011; Sionov *et al.* 2012; Flowers *et al.* 2015) occur in SRS causing azoles to interact and bind differently within Cyp51. Increased expression levels of *cyp51A* due to 34-, 46-, 53-, and 120-bp tandem repeats in the promoter have occurred in *A. fumigatus* leading to high levels of pan-azole resistance, which is resistance to more than 1 azole drug (Snelders *et al.* 2008; Hodiamont *et al.* 2009; Snelders *et al.* 2010; Hare *et al.* 2019). Tandem repeats in the *cyp51A* promoter reduce the affinity of the promoter and the CGAAT-binding complex, which binds to CGAAT in the promoter and downregulates *cyp51A* expression (Gsaller *et al.* 2016). Although Cyp51A has been the focus of most studies in *A. fumigatus*, a second paralog (Cyp51B) has also been documented to cause resistance through upregulation and missense mutations (Buied *et al.* 2013; Gonzalez-Jimenez *et al.* 2020). Like human pathogens, plant pathogens (such as *Erysiphe necator, Mycosphaerella* spp.*, Penicillium digitatum*, and *Venturia inaequalis*) undergo changes in the *cyp51A* promoter (substitutions, insertions, and duplications) and/or mutations in SRS to alter expression and binding of Cyp51A (Délye *et al.* 1997; Schnabel and Jones 2001; Ghosoph *et al.* 2007; Cañas-Gutiérrez *et al.* 2009; Stammler *et al.* 2009; Cools *et al.* 2012; Gadoury *et al.* 2012; Pfeufer and Ngugi 2012; Cools and Fraaije 2013; Sun *et al.* 2013).

Previous studies were limited to fewer than 86 fungal sequences (Hawkins *et al.* 2014; Song *et al.* 2018) or only to *Aspergillus* spp. (Dos Santos *et al.* 2020; Perez-Cantero *et al.* 2020). The goal of our study was to better understand the relationships among all fungal Cyp51 proteins.

## Materials and methods

### NCBI protein Blast

*Aspergillus fumigatus* Cyp51A protein sequence (XP_752137.1) was used in an NCBI Protein BLAST to search the reference sequence database for other Cyp51 proteins in Fungi. The following setting were used for the Protein BLAST: Database: Reference Proteins, Exclude: uncultured/environmental sample sequences, Algorithm: blastp (protein-protein BLAST), Max Target Sequences: 1000, Expect Threshold: 0.001, Word size: 6, Max matches in a query range: 0, Matrix: BLOSUM62, Gap Costs: Existence: 11 Extension: 1, and Compositional adjustments: Conditional compositional score matrix adjustment. Sequences with <50% coverage and <30% percent identity were eliminated. To ensure all clades were represented, the top hit from each clade was used to conduct another protein BLAST search (Supplementary Table 1). Some clades were not represented in the reference sequence database, so identical settings were used to search and filter the nonredundant protein sequences database. The unfiltered searches resulted in a total of 4,404 sequence hits. Sequences were filtered to have >50% coverage and more than 70% percent identity. FASTA files of the 480 sequences resulting from filtering were downloaded and opened in Geneious Prime 2019.1.1. To confirm the sequences were a Cyp51, sequences were checked for the presence of SRS1–6, the oxygen-binding motif AGXDTT, PER, and EXXR motifs, and the conserved FXXGXXXCXG heme-binding domain.

The following sequences were eliminated for missing amino acids in SRS1–6 or missing amino acids in conserved motifs: EPZ30787.1, EPZ31936.1, KXN68292.1, RKP07181.1, RKP16598.1, RKP17874.1, RKP18653.1, RKP18926.1, XP_001218650.1, XP_002563403.1, XP_002583031.1, XP_002842283.1, XP_003005233.1, XP_003325369.2, XP_007375289.1, XP_007756389.1, XP_007802603.1, XP_008039623.1, XP_009649122.1, XP_013258864.1, XP_015404015.1, XP_018230821.1, XP_018249826.1, XP_018270027.1, XP_018712692.1, XP_020066776.1, XP_022578172.1, XP_025599710.1, XP_027619241.1, XP_027619242.1, XP_031034290.1, XP_031059536.1, ORZ32486.1, XP_017991977.1, XP_003017020.1, XP_003019064.1, and XP_033461214.1. The following 19 fusion proteins were found in various members in Ascomycota: XP_022511803.1, XP_013278994.1, XP_024670717.1, XP_031899359.1, XP_031927262.1, XP_026621438.1, XP_025554838.1, XP_018192447.1, XP_031935516.1, XP_025433272.1, XP_015404994.1, XP_024709601.1, XP_007688940.1, XP_014073145.1, XP_025394250.1, XP_014555703.1, XP_007712864.1, XP_033384229.1, XP_018700143.1. *Aspergillus flavus* was reported to contain 3 Cyp51 proteins (Liu *et al.* 2012; Paul *et al.* 2015), but these were later removed by NCBI (https://www.ncbi.nlm.nih.gov/protein/XP_002383931.1/) (XP_002375123.1, XP_002379130.1, XP_002383931.1). Some *Aspergillus* spp. have a duplication of Cyp51A or Cyp51B which has previously been called Cyp51C (Perez-Cantero *et al.* 2020). NCBI refSeq is not exclusively based on whole genome sequences so it should be noted that it is possible that the full complement of Cyp51 orthologs in each of the 295 species was not identified.

### Phylogenetic analyses

Protein sequences were aligned once with MAFFT version 7.407 then once with PASTA version 1.8.5 (Katoh and Standley 2013; Mirarab *et al.* 2015). Maximum likelihood trees were constructed with RAxML version 8.2.11 with a PROTGAMMAAUTO or GTRGAMMA substitution model and 1000 bootstraps (Stamatakis 2014). Interactive Tree of Life (iTOL) was used for visualization and annotation of the trees (Letunic and Bork 2019). Species were represented by a single Cyp51 protein sequence. Subphylum Pezizomycotina was represented by group Cyp51B where duplicate Cyp51B sequences were removed.

### Amino acid analyses

Geneious Prime (version 2021.2.2) was used to generate pairwise identities and consensus sequences. Similarity tables for the whole protein and motifs are based on pairwise identity in each group, number of similar amino acids divided by the total number of amino acids in the protein or motif. “Weblogo-like” diagrams were created manually for visualization of conservation across groups. The height of 1 letter amino acid designation was based on frequency across all 4 consensus sequences. Colors and symbols were used as described in Fig. 5 to denote conservation within groups.

## Results and discussion

### Four hundred thirty-five fungal Cyp51 proteins were analyzed

Cyp51 proteins were previously defined as having 6 SRS, an oxygen-binding motif (AGXDTT), PER and EXXR motifs that create an E–R–R triad within the heme pocket, and a conserved heme-binding motif (FXXGXXXCXG) (Lepesheva and Waterman 2007; Córdova *et al.* 2017) (Fig. 1). To understand Cyp51 genes across Fungi, the Cyp51A protein from *A. fumigatus* strain Af293—a well-studied clinical strain used by multiple laboratories (Garcia-Rubio *et al.* 2018; Keizer *et al.* 2021)—was used as a reference in a protein Basic Local Alignment Search Tool (BLAST) (Altschul *et al.* 1990). A total of 4,404 protein sequences resulted and were filtered to retain those with >50% coverage and >30% percent identity to the reference sequence, resulting in 480 sequences (Supplementary Table 1). The resulting protein sequences were analyzed for the presence of full length SRS1–6 domains and the 4 Cyp51 motifs. Of these, 435 proteins had SRS1–6 domains, the oxygen-binding motif AGXDTT, the PER and EXXR motifs, and the conserved heme-binding motif FXXGXXXCXG and were considered to be functional Cyp51 proteins (Supplementary Table 1). Among the 435 Cyp51 sequences that met our criteria for inclusion, JGI and NCBI listed 15 apparent fusion proteins; 13 between Cyp51B and a kinase immediately upstream and 2 between Cyp51C and an acetyltransferase immediately upstream. To determine whether the 15 apparent fusion proteins were bona fide fusion proteins or 2 separate proteins merged by in silico errors, we searched syntenic regions in related species in FungiDB and MycoCosm (Grigoriev *et al.* 2012; Amos *et al.* 2022). Syntenic regions showed some separate proteins with intergenic regions between them, some overlapping proteins, and some fused proteins (Grigoriev *et al.* 2012; Amos *et al.* 2022). The Cyp51 portions of the fusion proteins were extracted and kept in the analyses resulting in a total of 435 Cyp51 proteins from 295 species (Supplementary Tables 1 and 2). It should be noted that our search parameters ensure that all 435 sequences we analyzed are true Cyp51 proteins; however, it is possible that the full complement of Cyp51 paralogs in each of the 295 species was not identified because refSeq is not exclusively based on whole genome sequences.

### Fungal Cyp51 proteins fall into 4 groups

**Fig. 2.**
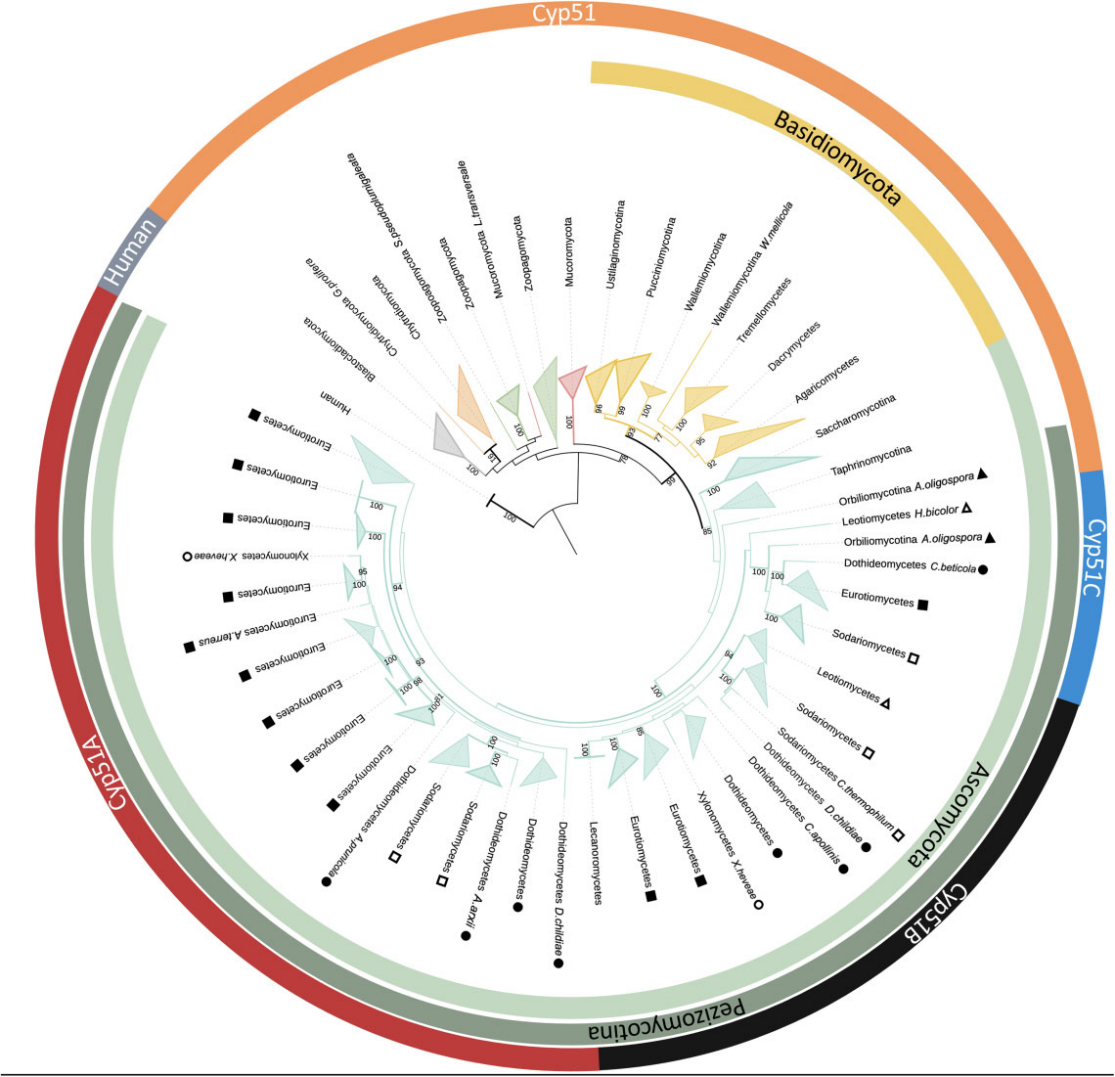
Cyp51 protein tree for fungi. Maximum likelihood tree of 435 Cyp51 proteins with collapsed branches based on phyla, subphyla, or classes. Branch colors match the colors used for taxonomic clades in the Fungal Tree of Life (James, Stajich, *et al.* 2020). Branches for Blastocladiomycota, Chytridiomycota, Monoblepharidomycota, Zoopagomycota, Mucoromycota, Basidiomycota, and Ascomycota are represented by gray, orange, blue, green, red, yellow, and teal, respectively. Branches with bootstrap support of at least 90 are in bold. Collapsed branches represent phyla, subphyla and classes and are named accordingly. Shapes represent subphyla and classes in Ascomycota. Filled triangles represent subphylum Orbiliomycotina. Empty triangles, filled circles, empty circles, filled squares, and empty squares represent classes Leotiomycetes, Dothideomycetes, Xylonomycetes, Eurotiomycetes, and Sodariomycetes, respectively. The innermost ring shows phyla Basidiomycota (yellow) and Ascomycota (teal). The second ring shows subphylum Pezizomycotina (dark teal). The outer ring shows human Cyp51 proteins used as the outgroup (grey) and 4 groups of fungal Cyp51 proteins—Cyp51, Cyp51C, Cyp51B, and Cyp51A represented by orange, blue, black and red, respectively.

To investigate how fungal Cyp51 proteins are related to each other, we used RAxML to create a maximum likelihood tree with 435 fungal Cyp51 proteins and 2 human Cyp51 proteins to root the tree (Supplementary Fig. 1). To aid in visualization, we collapsed branches based on phyla, subphyla, or classes (Fig. 2). We found fungal Cyp51 proteins fell into 4 major groups which we designated as Cyp51, Cyp51A, Cyp51B, and Cyp51C based on naming in previous literature (Fig. 2; Supplementary Fig. 1) (Hawkins *et al.* 2014; Perez-Cantero *et al.* 2020). Cyp51 in members of Saccharomycotina and Taphrinomycotina are also known as “Erg11” in the literature. The topology of our Cyp51 protein tree largely followed the topology of the fungal tree of life [Fig. 2; Supplementary Fig. 1 (James, Stajich, *et al.* 2020)]. Proteins from early-diverging fungi (Blastocladiomycota, Chytridiomycota, Monoblepharidomycota, Zoopagomycota, and Mucormycota), Basidiomycota, Saccharomycotina, and Taphrinomycotina fell into group Cyp51. Our phylogenetic analyses show a divergence of 3 Cyp51 paralogs in filamentous Ascomycota (Fig. 2; Supplementary Fig. 1). Those from Pezizomycotina fell into Cyp51A, Cyp51B, and Cyp51C. Divergence of Cyp51 from the common ancestor of paralogs Cyp51A, Cyp51B, and Cyp51C has strong support (100% bootstrap support), but divergence of paralogs Cyp51A, Cyp51B, and Cyp51C from each other does not have strong support (41%, 36%, and 41% bootstrap support, respectively).

**Fig. 3.**
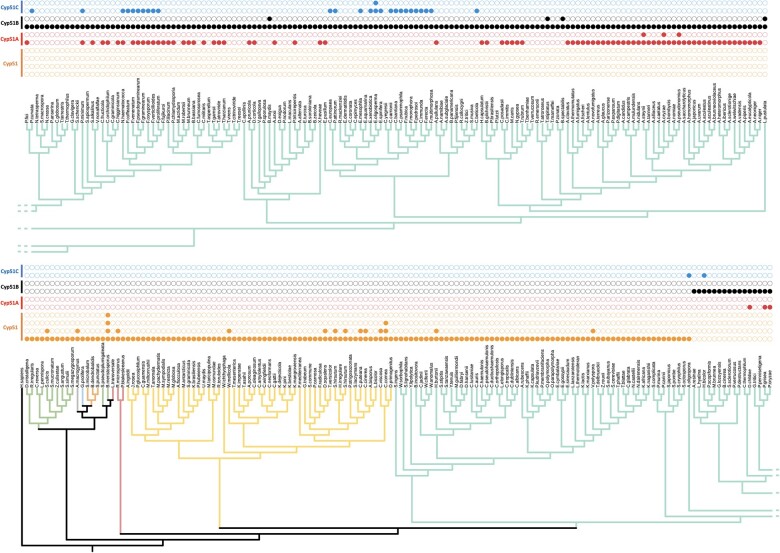
Fungal species have different numbers of Cyp51 paralogs. Overview of 435 Cyp51 paralogs in 295 fungal species based on Supplementary Table 2 and Supplementary Fig. 1 maximum likelihood tree. Orange, red, black, and blue circles show number of Cyp51, Cyp51A, Cyp51B, and Cyp51C paralogs, respectively. For ease of viewing, where multiple strains represent a species in the maximum likelihood tree, all paralogs were combined in a single entry for that species. Branch colors match the colors used for taxonomic clades in the Fungal Tree of Life (James, Stajich, *et al.* 2020). Branches for Blastocladiomycota, Chytridiomycota, Monoblepharidomycota, Zoopagomycota, Mucoromycota, Basidiomycota, and Ascomycota are represented by gray, orange, blue, green, red, yellow, and teal, respectively.

Most fungal species in our study had 1 or 2 Cyp51 paralogs (180/295 and 100/295, respectively) (Fig. 3; Supplementary Table 2). Fewer had 3 (14/295) and only 1 fungus, *Basidiobolus meristosporus*, had 4 copies of Cyp51 (Fig. 3). Fungi with 1 or 2 Cyp51 proteins were found across all taxonomic groups (Fig. 3). Fungi with 3 Cyp51 proteins were found in Basidiomycota and Ascomycota. Members in Pezizomycotina had different combinations of Cyp51 paralogs (Fig. 3). Most species had Cyp51A and Cyp51B paralogs or only Cyp51B (69/171 and 63/171, respectively) (Fig. 3). All members of Pezizomycotina contained a Cyp51B paralog (Fig. 3). As shown in Supplementary Table 2, we named fungal Cyp51 proteins based on the group assignment in Supplementary Fig. 1.

**Fig. 4.**
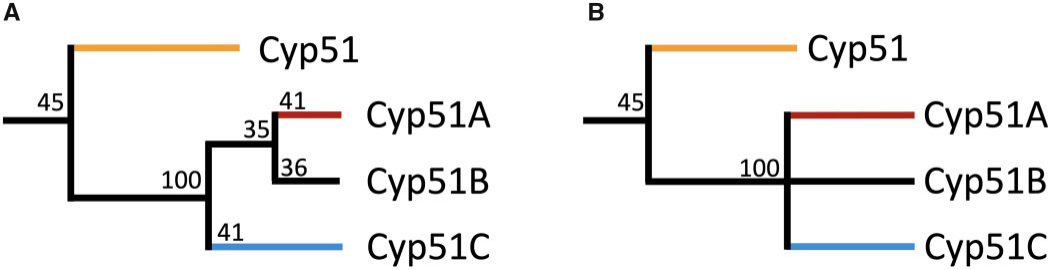
Possible Cyp51 homolog evolutionary paths. Simplified diagrams of possible Cyp51 evolutionary paths based on Supplementary Fig. 1 (A) and Fig. 2 (B). The Cyp51 branch in early-diverging fungi, Basidiomycota, Saccharomycotina, and Taphrinomycotina. Filamentous Cyp51A, Cyp51B, and Cyp51C branches represent Ascomycota (Pezizomycotina). Numbers represent bootstrap support.

We postulate 2 possible evolutionary paths for Cyp51 paralogs as shown in Fig. 4. In the first possible evolutionary path, shown in Fig. 4a, after an initial Cyp51 duplication paralog C diverged followed by another duplication event and divergence of paralogs A and B. The divergence of paralogs A (41%), B (36%), and C (41%) has low support. In the second possible evolutionary path, shown in Fig. 4b, the poorly supported nodes are removed so that the 3 paralogs diverged after 2 unresolved duplication events or a triplication event placing them on the same branch. In either scenario it is possible that subsequent gene loss(es) or duplication(s) led to species with the different combinations of paralogs shown in Fig. 3.

Distinguishing between these possible evolutionary paths is complicated by the small number of Cyp51C sequences and the relatively low number of characters (414–624 amino acids) in Cyp51 genes resulting in low bootstrap support for some nodes. Our analysis only had 29 Cyp51C sequences compared with Cyp51A and B with 87 and 171 sequences, respectively. To see if we could better resolve the relationships among Cyp51A, Cyp51B, and Cyp51C, we analyzed pairwise conservation of all Cyp51 protein sequences using Geneious Prime (Supplementary Table 3). We found that individual members of the Cyp51 group varied the most from each other, with only 46% similarity. Similarity within the Cyp51A, Cyp51B, and Cyp51C groups was much higher (64.7–68.8%). Comparing between groups, members of the Cyp51 group were 45–50% similar to members of Cyp51A, Cyp51B, or Cyp51C groups while members of Cyp51A, Cyp51B, and Cyp51C groups were roughly 60% similar to each other (Supplementary Table 3). We then examined conservation within the highly conserved motifs (SRS1–6, AGXDTT, PER, EXXR, and FXXGXXXCXG) (Supplementary Table 4). Once more the general trend was that motifs within the Cyp51 group were more variable that those in other groups.

**Fig. 5.**
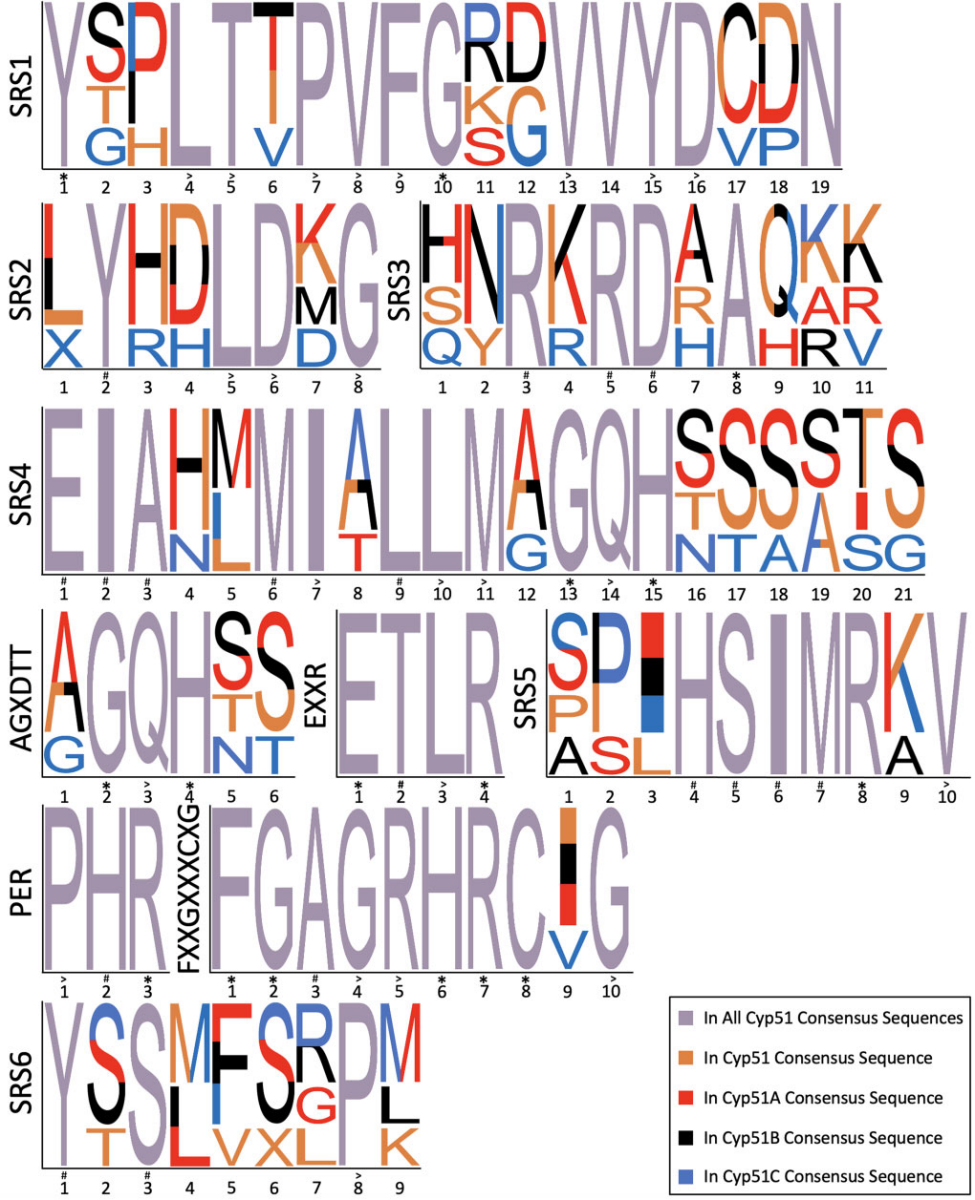
Conservation of motifs in Cyp51 proteins across Fungi. Consensus sequences for each of the 4 Cyp51A groups are shown arranged from the N- to C-terminus. Amino acids conserved across all 435 Cyp51 protein sequences are denoted by an *. Amino acids found in more than 95% of all Cyp51s are denoted by >. Lower than 95% conservation in all Cyp51s are denoted by #. Numbers represent amino acid position within motifs.

Consensus sequences from the 4 Cyp51 groups were compared with each other to create a visual representation of differences in motifs across groups (Fig. 5; Supplementary Table 4).

The most conserved regions (>95% across all Cyp51 proteins) are in the EXXR, PER, and FXXGXXXCIG motifs (Fig. 5; Supplementary Table 4). These 3 motifs are presumably highly conserved due to their roles in Cyp51 structure and function. The **E**XX**R** and PE**R** motif form the **E**–**R**–**R** triad that stabilizes the core structure of Cyp51, while the FXXGXXXCIG motif is a heme-binding domain that is essential for Cyp51 function (Deng *et al.* 2007; Sezutsu *et al.* 2013). Out of the 17 amino acids within these 3 motifs, 13 are more than 95% conserved (Fig. 5; Supplementary Table 4). Looking at all motifs, there are 59 amino acids shared between the 4 consensus sequences with 39 having >95% conservation (Fig. 5; Supplementary Table 4). Cyp51A and Cyp51B have the highest amount of shared amino acids in motifs (88%) (Fig. 5; Supplementary Table 4). Cyp51C has 23 amino acids across motifs that are unique compared with 15 in Cyp51, 8 in Cyp51A, and 5 in Cyp51B, suggesting Cyp51C may be a specialized group, though the low number of Cyp51C sequences included in the analysis might also explain the pattern (Fig. 5).

Research on Cyp51 in filamentous Ascomycota tends to be focused on the Cyp51A paralog because of its role in azole resistance in fungal pathogens of plants and animals. To our surprise, we found Cyp51A in only half of the species of filamentous Ascomycota we analyzed (86/171) and Cyp51B in all species (Fig. 3; Supplementary Table 2). The low bootstrap support we saw for divergence of Cyp51A from Cyp51B suggests they may play very similar roles with Cyp51B being the essential paralog and the possible Cyp51 ortholog (Fig. 4a). Indeed, in *A. fumigatus*, Cyp51A and Cyp51B act in a compensatory manner; when one paralog is knocked out expression of the other paralog increases (Roundtree *et al.* 2020). Our results confirm and expand previous work by Hawkins *et al.* (2014) that compared 86 fungal Cyp51 proteins in 54 different species and found all species of filamentous Ascomycota retained a Cyp51B paralog, but Cyp51A had been lost in multiple lineages, and Cyp51C was only found in *Fusarium* spp. (Becher *et al.* 2011; Hawkins *et al.* 2014; James, Lamping, *et al.* 2020). Previous publications have identified Cyp51C in *A. flavus* (Liu *et al.* 2012; Paul *et al.* 2015)*;* however, these sequences were removed from NCBI (https://www.ncbi.nlm.nih.gov/protein/XP_002383931.1/). Other *Aspergillus* spp. have been reported to have a Cyp51C, but these were a duplication of Cyp51A or Cyp51B (Perez-Cantero *et al.* 2020). Cyp51C was subsequently reported in *Fusarium* spp., *Gibberella zeae*, and *Nectria haematoccoa* (Becher *et al.* 2011; Hawkins *et al.* 2014)*.* We found Cyp51C in 9 other genera (Supplementary Table 2)*.* Interestingly, all are pathogens of plants or animals (Supplementary Table 2). Cyp51C has been shown to be necessary for invasion of plant host tissues in *Fusarium* (Fan *et al.* 2013)*.* This raises the interesting possibility that it could play a similar role in other genera, though further functional studies are needed to test the role of Cyp51C in invasion and virulence.

Maximum likelihood analysis suggested 2 possible evolutionary paths for Cyp51 paralogs: (1) Cyp51C diverged before Cyp51A and Cyp51B (Fig. 4a); (2) Cyp51A, Cyp51B, and Cyp51C diverged from each other at the same time (Fig. 4b). Based on the higher amino acid conservation between Cyp51A and Cyp51B, the evolutionary path shown in Fig. 4a seems more likely; that is to say Cyp51C is a specialized group of proteins that diverged first (Figs. 4a and 5; Supplementary Table 4). More functional studies are needed to understand shared and unique roles Cyp51A, Cyp51B, and Cyp51C paralogs play in fungi.

## Supplementary Material

jkac249_Supplemental_Figure_1_Legend

jkac249_Figure_S1

jkac249_Supplemental_Table_S1

jkac249_Supplemental_Table_S2

jkac249_Supplemental_Table_S3

jkac249_Supplemental_Table_S4

## Data Availability

All Cyp51 sequences used are listed in Supplementary Table 2 and are publicly available through NCBI (https://www.ncbi.nlm.nih.gov/). Supplemental material is available at G3 online.
