# Supplementary material for: Analysis of Cyp51 protein sequences shows 4 major Cyp51 gene family groups across fungi
Source: G3 (Bethesda). 2022 Sep 21;12(11):jkac249. doi: 10.1093/g3journal/jkac249 (PMC9635630; doi:10.1093/g3journal/jkac249)
Supplement: jkac249_Supplemental_Figure_1_Legend [file jkac249_supplemental_figure_1_legend.docx]

**Supplemental Figure 1: Cyp51 protein tree for Fungi.** Maximum likelihood tree of 435 fungal Cyp51 proteins. Branch colors match the colors used for taxonomic clades in the Fungal Tree of Life [[47](#_ENREF_36)]. Branches for Blastocladiomycota, Chytridiomycota, Zoopagomycota, Mucoromycota, Basidiomycota, and Ascomycota are represented by gray, orange, blue, green, red, yellow, and teal, respectively. Branches with bootstrap support of at least 90 are in bold. The bar to the right shows human Cyp51 proteins used as the outgroup (grey) and 4 groups of fungal Cyp51 proteins – Cyp51, Cyp51C, Cyp51B, and Cyp51A represented by orange, blue, black and red, respectively.
