# Supplementary material for: Analysis of Cyp51 protein sequences shows 4 major Cyp51 gene family groups across fungi
Source: G3 (Bethesda). 2022 Sep 21;12(11):jkac249. doi: 10.1093/g3journal/jkac249 (PMC9635630; doi:10.1093/g3journal/jkac249)
Supplement: jkac249_Supplemental_Table_S1 [file jkac249_supplemental_table_s1.docx]

**Supplemental Table 1. Summary of BLAST results for each fungal clade.​**

| Clade^1^​ | Query Sequence​ | Hits Before Filter^3^​ | Hits After Filter^4^ | Met Cyp51 Criteria​^5^ | Number of Species^6^ |
| --- | --- | --- | --- | --- | --- |
| Agaricomycetes​ | XP_006955920.1​ | 543​ | 23​ | 20 | 15 |
| Blastocladiomycota^2^​ | XP_016612382.1​ | 69​ | 3​ | 2​ | 1 |
| Chytridiomycetes​ | XP_025190100.1​ | 34​ | 3​ | 3 | 3 |
| Cryptomycota^2^​ | XP_01661282.1​ | 8​ | 8​ | 0​ | 0 |
| Dacrymycetes^2^​ | XP_018263285.1​ | 101​ | 6​ | 6​ | 3 |
| Dothideomycetes​ | XP_752137.1​ | 554​ | 34​ | 32 | 25 |
| Eurotiomycetes​ | XP_018249823.1​ | 1012​ | 163​ | 151 | 80 |
| Glomeromycotina​ | XP_018226934.1​ | 56​ | 1​ | 1​ | 1 |
| Lecanoromycetes^2^​ | XP_752137.1​ | 37​ | 2​ | 2​ | 1 |
| Leotiomycetes​ | XP_752137.1​ | 205​ | 11​ | 11​ | 10 |
| Microsporidia^2^​ | XP_01661282.1​ | NA​ | NA​ | NA​ | NA |
| Monoblepharidomycetes^2^​ | XP_016612382.1​ | 32​ | 1​ | 1​ | 1 |
| Mortierellomycotina​ | XP_025190100.1​ | 8​ | 1​ | 1​ | 1 |
| Mucoromycotina​ | XP_025190100.1​ | 30​ | 3​ | 3​ | 2 |
| Neocallimastigomycota^2^​ | XP_016612382.1​ | NA​ | NA​ | NA​ | NA |
| Orbiliomycetes​ | XP_752137.1​ | 10​ | 2​ | 2​ | 1 |
| Pezizomycetes​ | XP_752137.1​ | 6​ | 1​ | 0​ | 0 |
| Pucciniomycotina​ | XP_006955920.1​ | 23​ | 5​ | 3​ | 3 |
| Saccharomycotina​ | XP_752137.1​ | 231​ | 56​ | 53 | 51 |
| Sordariomycetes​ | XP_752137.1​ | 1020​ | 96​ | 88 | 48 |
| Taphrinomycotina​ | XP_015469227.1​ | 16​ | 8​ | 7 | 7 |
| Tremellomycetes​ | XP_006955920.1​ | 48​ | 14​ | 14​ | 13 |
| Ustilaginomycotina​ | XP_006955920.1​ | 102​ | 17​ | 15 | 15 |
| Wallemiomycetes​ | XP_018263205.1​ | 7​ | 3​ | 3​ | 2 |
| Xylonomycetes​ | XP_752137.1​ | 8​ | 2​ | 2​ | 1 |
| Zoopagomycetes^2^​ | XP_016612382.1​ | 244​ | 17​ | 15​ | 11 |
| Total​ | | 4404​ | 480​ | 435 | 295 |

^1^The clades Entomorphthoromycotina and Kickxellomycotina are not shown in the table as they were not represented within the reference protein sequences nor the non-redundant protein sequences databases.

^2^These clades were not represented in the reference protein sequences database, but hits were found within the non-redundant protein sequences database.

^3^Search parameters used: Database: Reference Proteins, Exclude: uncultured/environmental sample sequences, Algorithm: blastp (protein-protein BLAST), Max Target Sequences: 1000, Expect Threshold: 0.001, Word size: 6, Max matches in a query range: 0, Matrix: BLOSUM62, Gap Costs: Existence: 11 Extension: 1, and Compositional adjustments: Conditional compositional score matrix adjustment

^4^Filters were set to eliminate sequences with less than 50% coverage and less than 30% percent identity.

^5^A protein was considered a Cyp51 if it had full length SRS1-6 domains and the four Cyp51 motifs.

^6^Number of species with Cyp51 proteins that passed filtering criteria.
