# Supplementary material for: Analysis of Cyp51 protein sequences shows 4 major Cyp51 gene family groups across fungi
Source: G3 (Bethesda). 2022 Sep 21;12(11):jkac249. doi: 10.1093/g3journal/jkac249 (PMC9635630; doi:10.1093/g3journal/jkac249)
Supplement: jkac249_Supplemental_Table_S2 [file jkac249_supplemental_table_s2.docx]

**Supplemental Table 2: 435 Fungal Cyp51s.** Protein BLAST results after filtering for greater than 50% coverage, greater than 30% percent identity to the *A. fumigatus* Cyp51A reference, and meeting Cyp51 criteria. Accession numbers, current name, phylum and species names are based on NCBI records for each Cyp51. Tip labels are based on species name and clade. Clade/proposed name is based on clade designation from Supplemental Figure 1. Phyla are abbreviated by the first two letters: As, Ascomycota; Ba, Basidiomycota; Bl, Blastocladiomycota; Ch, Chytridiomycota; Mo, Monoblepharidomycota; Mu, Mucoromycota; Zo, Zoopagomycota.

| **Accession #**^1^ | **Tip Label**^2^ | **Clade**^3^ | **Current Name**^4^ | **Phylum**^5^ | **Proposed Name**^6^ | **Species/Strain**^7^ |
| --- | --- | --- | --- | --- | --- | --- |
| XP_033388152.1 | A.arxii_Cyp51A | Cyp51A | NA | As | Cyp51A | *Aaosphaeria arxii* CBS 175.79 |
| XP_025375855.1 | A.ingoldii_Cyp51 | Cyp51 | NA | Ba | Cyp51 | *Acaromyces ingoldii* |
| XP_006459056.1 | A.bisporusH97_Cyp51 | Cyp51 | NA | Ba | Cyp51 | *Agaricus bisporus* var. bisporus H97 |
| XP_007331417.1 | A.bisporusJB137S8_Cyp51 | Cyp51 | NA | Ba | Cyp51 | *Agaricus bisporus* var. burnettii JB137-S8 |
| KNE72323.1 | A.macrogynus_Cyp51.1 | Cyp51 | NA | Bl | Cyp51 | *Allomyces macrogynus* ATCC 38327 |
| KNE68169.1 | A.macrogynus_Cyp51.2 | Cyp51 | NA | Bl | Cyp51 | *Allomyces macrogynus* ATCC 38327 |
| XP_018386596.1 | A.alternata_Cyp51B | Cyp51B | NA | As | Cyp51B | *Alternaria alternata* |
| XP_024720485.1 | A.resinae_Cyp51B | Cyp51B | NA | As | Cyp51B | *Amorphotheca resinae* ATCC 22711 |
| XP_007876928.1 | A.flocculosa_Cyp51 | Cyp51 | NA | Ba | Cyp51 | *Anthracocystis flocculosa* PF-1 |
| XP_028475600.1 | A.porosum_Cyp51 | Cyp51 | NA | Ba | Cyp51 | *Apiotrichum porosum* |
| XP_033402105.1 | A.prunicola_Cyp51A | Cyp51A | NA | As | Cyp51A | *Aplosporella prunicola* CBS 121167 |
| XP_033397510.1 | A.prunicola_Cyp51B | Cyp51B | NA | As | Cyp51B | *Aplosporella prunicola* CBS 121167 |
| XP_011121086.1 | A.oligospora_Cyp51 | Cyp51 | NA | As | Cyp51 | *Arthrobotrys oligospora* ATCC 24927 |
| XP_011122991.1 | A.oligospora_Cyp51C | Cyp51C | NA | As | Cyp51C | *Arthrobotrys oligospora* ATCC 24927 |
| XP_020048742.1 | A.rubescens_Cyp51 | Cyp51 | NA | As | Cyp51 | *Ascoidea rubescens* DSM 1968 |
| XP_025508294.1 | A.aculeatinus_Cyp51A | Cyp51A | NA | As | Cyp51A | *Aspergillus aculeatinus* CBS 121060 |
| XP_025502849.1 | A.aculeatinus_Cyp51B | Cyp51B | Cyp51B | As | Cyp51B^8^ | *Aspergillus aculeatinus* CBS 121060 |
| XP_020058751.1 | A.aculeatus_Cyp51A | Cyp51A | NA | As | Cyp51A | *Aspergillus aculeatus* ATCC 16872 |
| XP_020061717.1 | A.aculeatus_Cyp51B | Cyp51B | NA | As | Cyp51B | *Aspergillus aculeatus* ATCC 16872 |
| XP_031901970.1 | A.alliaceus_Cyp51A | Cyp51A | NA | As | Cyp51A | *Aspergillus alliaceus* |
| XP_031899359.1 | A.alliaceus_Cyp51B | Cyp51B | NA | As | Cyp51B | *Aspergillus alliaceus* |
| XP_022394184.1 | A.bombycis_Cyp51A | Cyp51A | NA | As | Cyp51A | *Aspergillus bombycis* |
| XP_022386569.1 | A.bombycis_Cyp51B | Cyp51B | NA | As | Cyp51B | *Aspergillus bombycis* |
| XP_025438807.1 | A.brunneoviolaceus_Cyp51A | Cyp51A | NA | As | Cyp51A | *Aspergillus brunneoviolaceus* CBS 621.78 |
| XP_025437311.1 | A.brunneoviolaceus_Cyp51B | Cyp51B | Cyp51B | As | Cyp51B^8^ | *Aspergillus brunneoviolaceus* CBS 621.78 |
| XP_031923728.1 | A.caelatus_Cyp51A | Cyp51A | NA | As | Cyp51A | *Aspergillus caelatus* |
| XP_031927262.1 | A.caelatus_Cyp51B | Cyp51B | NA | As | Cyp51B | *Aspergillus caelatus* |
| XP_024691801.1 | A.campestris_Cyp51A | Cyp51A | NA | As | Cyp51A | *Aspergillus campestris* IBT 28561 |
| XP_024689915.1 | A.campestris_Cyp51B | Cyp51B | Cyp51B | As | Cyp51B^8^ | *Aspergillus campestris* IBT 28561 |
| XP_024675268.1 | A.candidus_Cyp51A | Cyp51A | NA | As | Cyp51A | *Aspergillus candidus* |
| XP_024670717.1 | A.candidus_Cyp51B | Cyp51B | NA | As | Cyp51B | *Aspergillus candidus* |
| XP_001271579.1 | A.clavatus_Cyp51A | Cyp51A | Cyp51A | As | Cyp51A^8^ | *Aspergillus clavatus* NRRL 1 |
| XP_001273214.1 | A.clavatus_Cyp51B | Cyp51B | Cyp51B | As | Cyp51B^8^ | *Aspergillus clavatus* NRRL 1 |
| XP_025536274.1 | A.costaricaensis_Cyp51A | Cyp51A | Cyp51A | As | Cyp51A^8^ | *Aspergillus costaricaensis* CBS 115574 |
| XP_025538767.1 | A.costaricaensis_Cyp51B | Cyp51B | Cyp51B | As | Cyp51B^8^ | *Aspergillus costaricaensis* CBS 115574 |
| XP_025383643.1 | A.eucalypticola_Cyp51A | Cyp51A | NA | As | Cyp51A | *Aspergillus eucalypticola* CBS 122712 |
| XP_025385603.1 | A.eucalypticola_Cyp51B | Cyp51B | Cyp51B | As | Cyp51B^8^ | *Aspergillus eucalypticola* CBS 122712 |
| XP_001267338.1 | A.fischeri_Cyp51A | Cyp51A | Cyp51A | As | Cyp51A^8^ | *Aspergillus fischeri* NRRL 181 |
| XP_001261295.1 | A.fischeri_Cyp51B | Cyp51B | Cyp51B | As | Cyp51B^8^ | *Aspergillus fischeri* NRRL 181 |
| XP_752137.1 | A.fumigatus_Cyp51A | Cyp51A | Cyp51A | As | Cyp51A^8^ | *Aspergillus fumigatus* Af293 |
| XP_749134.1 | A.fumigatus_Cyp51B | Cyp51B | Cyp51B | As | Cyp51B^8^ | *Aspergillus fumigatus* Af293 |
| XP_022400916.1 | A.glaucus_Cyp51A | Cyp51A | NA | As | Cyp51A | *Aspergillus glaucus* CBS 516.65 |
| XP_022397807.1 | A.glaucus_Cyp51B | Cyp51B | NA | As | Cyp51B | *Aspergillus glaucus* CBS 516.65 |
| XP_025398211.1 | A.heteromorphus_Cyp51A | Cyp51A | Cyp51A | As | Cyp51A^8^ | *Aspergillus heteromorphus* CBS 117.55 |
| XP_025394250.1 | A.heteromorphus_Cyp51B | Cyp51B | NA | As | Cyp51B | *Aspergillus heteromorphus* CBS 117.55 |
| XP_025555526.1 | A.homomorphus_Cyp51A | Cyp51A | Cyp51A | As | Cyp51A^8^ | *Aspergillus homomorphus* CBS 101889 |
| XP_025554838.1 | A.homomorphus_Cyp51B | Cyp51B | NA | As | Cyp51B | *Aspergillus homomorphus* CBS 101889 |
| XP_025572098.1 | A.ibericus_Cyp51A | Cyp51A | Cyp51A | As | Cyp51A^8^ | *Aspergillus ibericus* CBS 121593 |
| XP_025580611.1 | A.ibericus_Cyp51B | Cyp51B | Cyp51B | As | Cyp51B^8^ | *Aspergillus ibericus* CBS 121593 |
| XP_025525178.1 | A.japonicus_Cyp51A | Cyp51A | NA | As | Cyp51A | *Aspergillus japonicus* CBS 114.51 |
| XP_025532422.1 | A.japonicus_Cyp51B | Cyp51B | Cyp51B | As | Cyp51B^8^ | *Aspergillus japonicus* CBS 114.51 |
| XP_033411287.1 | A.lentulus_Cyp51A | Cyp51A | NA | As | Cyp51A | *Aspergillus lentulus* |
| XP_033419929.1 | A.lentulus_Cyp51B | Cyp51B | NA | As | Cyp51B | *Aspergillus lentulus* |
| XP_026604222.1 | A.mulundensis_Cyp51A | Cyp51A | NA | As | Cyp51A | *Aspergillus mulundensis* |
| XP_026608416.1 | A.mulundensis_Cyp51B | Cyp51B | NA | As | Cyp51B | Aspergillus mulundensis |
| XP_025479801.1 | A.neoniger_Cyp51A | Cyp51A | Cyp51A | As | Cyp51A^8^ | *Aspergillus neoniger* CBS 115656 |
| XP_025473811.1 | A.neoniger_Cyp51B | Cyp51B | NA | As | Cyp51B | *Aspergillus neoniger* CBS 115656 |
| XP_659505.1 | A.nidulans_Cyp51A | Cyp51A | NA | As | Cyp51A | *Aspergillus nidulans* FGSC A4 |
| XP_681552.1 | A.nidulans_Cyp51B | Cyp51B | NA | As | Cyp51B | *Aspergillus nidulans* FGSC A4 |
| XP_025454722.1 | A.niger.101883_Cyp51A | Cyp51A | NA | As | Cyp51A | *Aspergillus niger* CBS 101883 |
| XP_025458281.1 | A.niger.101883_Cyp51B | Cyp51B | NA | As | Cyp51B | *Aspergillus niger* CBS 101883 |
| XP_001394224.1 | A.niger.513.88_Cyp51A | Cyp51A | NA | As | Cyp51A | *Aspergillus niger* CBS 513.88 |
| XP_001396151.2 | A.niger.513.88_Cyp51B | Cyp51B | NA | As | Cyp51B | *Aspergillus niger* CBS 513.88 |
| XP_015411243.1 | A.nomiae_Cyp51A | Cyp51A | NA | As | Cyp51A | *Aspergillus nomiae* NRRL 13137 |
| XP_015404994.1 | A.nomiae_Cyp51B | Cyp51B | NA | As | Cyp51B | *Aspergillus nomiae* NRRL 13137 |
| XP_024681713.1 | A.novofumigatus_Cyp51A | Cyp51A | Cyp51A | As | Cyp51A^8^ | *Aspergillus novofumigatus* IBT 16806 |
| XP_024679469.1 | A.novofumigatus_Cyp51B | Cyp51B | Cyp51B | As | Cyp51B^8^ | *Aspergillus novofumigatus* IBT 16806 |
| XP_001819419.1 | A.oryzae_Cyp51A.1 | Cyp51A | NA | As | Cyp51A | *Aspergillus oryzae* RIB40 |
| XP_001824687.1 | A.oryzae_Cyp51A.2 | Cyp51A | NA | As | Cyp51A | *Aspergillus oryzae* RIB40 |
| XP_001822241.1 | A.oryzae_Cyp51B | Cyp51B | NA | As | Cyp51B | *Aspergillus oryzae* RIB40 |
| XP_025515450.1 | A.piperis_Cyp51A | Cyp51A | NA | As | Cyp51A | *Aspergillus piperis* CBS 112811 |
| XP_025520356.1 | A.piperis_Cyp51B | Cyp51B | Cyp51B | As | Cyp51B^8^ | *Aspergillus piperis* CBS 112811 |
| XP_031947002.1 | A.pseudonomius_Cyp51A.1 | Cyp51A | NA | As | Cyp51A | *Aspergillus pseudonomius* |
| XP_031945788.1 | A.pseudonomius_Cyp51A.2 | Cyp51A | NA | As | Cyp51A | *Aspergillus pseudonomius* |
| XP_031935516.1 | A.pseudonomius_Cyp51B | Cyp51B | NA | As | Cyp51B | *Aspergillus pseudonomius* |
| XP_025431069.1 | A.saccharolyticus_Cyp51A | Cyp51A | Cyp51A | As | Cyp51A^8^ | *Aspergillus saccharolyticus* JOP 1030-1 |
| XP_025433272.1 | A.saccharolyticus_Cyp51B | Cyp51B | NA | As | Cyp51B | *Aspergillus saccharolyticus* JOP 1030-1 |
| XP_025469640.1 | A.sclerotioniger_Cyp51A | Cyp51A | Cyp51A | As | Cyp51A^8^ | *Aspergillus sclerotioniger* CBS 115572 |
| XP_025464341.1 | A.sclerotioniger_Cyp51B | Cyp51B | Cyp51B | As | Cyp51B^8^ | *Aspergillus sclerotioniger* CBS 115572 |
| XP_024710861.1 | A.steynii_Cyp51A.1 | Cyp51A | Cyp51A | As | Cyp51A^8^ | *Aspergillus steynii* IBT 23096 |
| XP_024701477.1 | A.steynii_Cyp51A.2 | Cyp51A | NA | As | Cyp51A | *Aspergillus steynii* IBT 23096 |
| XP_024709601.1 | A.steynii_Cyp51B | Cyp51B | NA | As | Cyp51B | *Aspergillus steynii* IBT 23096 |
| XP_033423673.1 | A.tanneri_Cyp51A | Cyp51A | NA | As | Cyp51A | *Aspergillus tanneri* |
| XP_033421528.1 | A.tanneri_Cyp51B | Cyp51B | NA | As | Cyp51B | *Aspergillus tanneri* |
| XP_001215095.1 | A.terreus_Cyp51A | Cyp51A | NA | As | Cyp51A | *Aspergillus terreus* NIH2624 |
| XP_001212028.1 | A.terreus_Cyp51B | Cyp51B | NA | As | Cyp51B | *Aspergillus terreus* NIH2624 |
| XP_026613844.1 | A.thermomutatus_Cyp51A | Cyp51A | NA | As | Cyp51A | *Aspergillus thermomutatus* |
| XP_026611814.1 | A.thermomutatus_Cyp51B | Cyp51B | NA | As | Cyp51B | *Aspergillus thermomutatus* |
| XP_025488378.1 | A.uvarum_Cyp51A | Cyp51A | Cyp51A | As | Cyp51A^8^ | *Aspergillus uvarum* CBS 121591 |
| XP_025491603.1 | A.uvarum_Cyp51B | Cyp51B | Cyp51B | As | Cyp51B^8^ | *Aspergillus uvarum* CBS 121591 |
| XP_025559392.1 | A.vadensis_Cyp51A | Cyp51A | NA | As | Cyp51A | *Aspergillus vadensis* CBS 113365 |
| XP_025565720.1 | A.vadensis_Cyp51B | Cyp51B | Cyp51B | As | Cyp51B^8^ | *Aspergillus vadensis* CBS 113365 |
| XP_026629604.1 | A.welwitschiae_Cyp51A | Cyp51A | Cyp51A | As | Cyp51A^8^ | *Aspergillus welwitschiae* |
| XP_026621438.1 | A.welwitschiae_Cyp51B | Cyp51B | NA | As | Cyp51B | *Aspergillus welwitschiae* |
| XP_013430052.1 | A.namibiae_Cyp51B | Cyp51B | Cyp51B | As | Cyp51B^8^ | *Aureobasidium namibiae* CBS 147.97 |
| XP_029756645.1 | A.pullulans_Cyp51A | Cyp51A | Cyp51A | As | Cyp51A^8^ | *Aureobasidium pullulans* EXF-150 |
| XP_029761237.1 | A.pullulans_Cyp51B | Cyp51B | Cyp51B | As | Cyp51B^8^ | *Aureobasidium pullulans* EXF-150 |
| XP_013341833.1 | A.subglaciale_Cyp51B | Cyp51B | NA | As | Cyp51B | *Aureobasidium subglaciale* EXF-2481 |
| XP**_**018985355.1 | B.inositovora_Cyp51 | Cyp51 | NA | As | Cyp51 | *Babjeviella inositovora* NRRL Y-12698 |
| ORX98854.1 | B.meristosporus_Cyp51.2 | Cyp51 | NA | Zo | Cyp51 | *Basidiobolus meristosporus* CBS 931.73 |
| ORX82040.1 | B.meristosporus_Cyp51.3 | Cyp51 | NA | Zo | Cyp51 | *Basidiobolus meristosporus* CBS 931.73 |
| ORY07241.1 | B.meristosporus_Cyp51.4 | Cyp51 | NA | Zo | Cyp51 | *Basidiobolus meristosporus* CBS 931.73 |
| ORX92671.1 | B.meristosporus_Cyp51.1 | Cyp51 | Cyp51B | Zo | Cyp51^9^ | *Basidiobolus meristosporus* CBS 931.73 |
| XP_006681973.1 | B.dendrobatidis_Cyp51 | Cyp51 | NA | Ch | Cyp51 | *Batrachochytrium dendrobatidis* JAM81 |
| XP_007681629.1 | B.panamericana_Cyp51B | Cyp51B | NA | As | Cyp51B | *Baudoinia panamericana* UAMH 10762 |
| XP_008600143.1 | B.bassiana_Cyp51A | Cyp51A | Cyp51F2 | As | Cyp51A^9^ | *Beauveria bassiana* ARSEF 2860 |
| XP_008593540.1 | B.bassiana_Cyp51B | Cyp51B | Cyp51F1 | As | Cyp51B^9^ | *Beauveria bassiana* ARSEF 2860 |
| XP_014077539.1 | B.maydis_Cyp51B.1 | Cyp51B | NA | As | Cyp51B | *Bipolaris maydis* ATCC 48331 |
| XP_014073145.1 | B.maydis_Cyp51B.2 | Cyp51B | NA | As | Cyp51B | *Bipolaris maydis* ATCC 48331 |
| XP_007702075.1 | B.sorokiniana_Cyp51B | Cyp51B | NA | As | Cyp51B | *Bipolaris sorokiniana* ND90Pr |
| XP_007712864.1 | B.zeicola_Cyp51B | Cyp51B | NA | As | Cyp51B | *Bipolaris zeicola* 26-R-13 |
| XP_002624879.2 | B.gilchristii_Cyp51A | Cyp51A | NA | As | Cyp51A | *Blastomyces gilchristii* SLH14081 |
| XP_002627007.1 | B.gilchristii_Cyp51B | Cyp51B | NA | As | Cyp51B | *Blastomyces gilchristii* SLH14081 |
| XP_001549961.1 | B.cinerea_Cyp51B | Cyp51B | BcCyp51 | As | Cyp51B^9^ | *Botrytis cinerea* B05.10 |
| XP_028484171.1 | B.spectabilis_Cyp51B.1 | Cyp51B | NA | As | Cyp51B | *Byssochlamys spectabilis* |
| XP_028482752.1 | B.spectabilis_Cyp51B.2 | Cyp51B | Cyp51B | As | Cyp51B^8^ | *Byssochlamys spectabilis* |
| KZT52203.1 | C.cornea_Cyp51.1 | Cyp51 | NA | Ba | Cyp51 | *Calocera cornea* HHB12733 |
| KZT52204.1 | C.cornea_Cyp51.2 | Cyp51 | NA | Ba | Cyp51 | *Calocera cornea* HHB12733 |
| KZT52205.1 | C.cornea_Cyp51.3 | Cyp51 | NA | Ba | Cyp51 | *Calocera cornea* HHB12733 |
| KZP00505.1 | C.viscosa_Cyp51.1 | Cyp51 | NA | Ba | Cyp51 | *Calocera viscosa* TUFC12733 |
| KZP00504.1 | C.viscosa_Cyp51.2 | Cyp51 | NA | Ba | Cyp51 | *Calocera viscosa* TUFC12733 |
| XP_716761.1 | C.albicans_Cyp51 | Cyp51 | NA | As | Cyp51 | *Candida albicans* SC5314 |
| XP_028891800.1 | C.auris_Cyp51 | Cyp51 | NA | As | Cyp51 | *Candida auris* |
| XP_002420370.1 | C.dubliniensis_Cyp51 | Cyp51 | NA | As | Cyp51 | *Candida dubliniensis* CD36 |
| XP_025336625.1 | C.duobushaemulonis_Cyp51 | Cyp51 | NA | As | Cyp51 | *Candida duobushaemulonis* |
| XP_445876.1 | C.glabrata_Cyp51 | Cyp51 | NA | As | Cyp51 | *Candida glabrata* |
| XP_025344294.1 | C.haemulonis_Cyp51 | Cyp51 | NA | As | Cyp51 | *Candida haemulonis* |
| XP_003870303.1 | C.orthopsilosis_Cyp51 | Cyp51 | Erg11 | As | Cyp51/Erg11^9^ | *Candida orthopsilosis* Co 90-125 |
| XP_024711630.1 | C.pseudohaemulonis_Cyp51 | Cyp51 | NA | As | Cyp51 | *Candida pseudohaemulonis* |
| XP_002550985.1 | C.tropicalis_Cyp51 | Cyp51 | NA | As | Cyp51 | *Candida tropicalis* MYA-3404 |
| XP_007720659.1 | C.coronata_Cyp51B | Cyp51B | NA | As | Cyp51B | *Capronia coronata* CBS 617.96 |
| XP_007736805.1 | C.epimyces_Cyp51B | Cyp51B | NA | As | Cyp51B | *Capronia epimyces* CBS 606.96 |
| XP_025371928.1 | C.guamensis_Cyp51 | Cyp51 | NA | Ba | Cyp51 | *Ceraceosorus guamensis* |
| XP_023450255.1 | C.beticola_Cyp51B | Cyp51B | NA | As | Cyp51B | *Cercospora beticola* |
| XP_023452197.1 | C.beticola_Cyp51C | Cyp51C | NA | As | Cyp51C | *Cercospora beticola* |
| XP_001220873.1 | C.globosum_Cyp51B | Cyp51B | NA | As | Cyp51B | *Chaetomium globosum* CBS 148.51 |
| XP_006696132.1 | C.thermophilum_Cyp51B | Cyp51B | NA | As | Cyp51B | *Chaetomium thermophilum* var. thermophilum DSM 1495 |
| XP_016621098.1 | C.bantiana_Cyp51B | Cyp51B | NA | As | Cyp51B | *Cladophialophora bantiana* CBS 173.52 |
| XP_016615967.1 | C.bantiana_Cyp51C | Cyp51C | NA | As | Cyp51C | *Cladophialophora bantiana* CBS 173.52 |
| XP_008724187.1 | C.carrionii_Cyp51B | Cyp51B | NA | As | Cyp51B | *Cladophialophora carrionii* CBS 160.54 |
| XP_008727072.1 | C.carrionii_Cyp51C | Cyp51C | NA | As | Cyp51C | *Cladophialophora carrionii* CBS 160.54 |
| XP_016246563.1 | C.immunda_Cyp51B | Cyp51B | NA | As | Cyp51B | *Cladophialophora immunda* |
| XP_016248307.1 | C.immunda_Cyp51C | Cyp51C | NA | As | Cyp51C | *Cladophialophora immunda* |
| XP_007751747.1 | C.psammophila_Cyp51B | Cyp51B | NA | As | Cyp51B | *Cladophialophora psammophila* CBS 110553 |
| XP_007742105.1 | C.psammophila_Cyp51C | Cyp51C | NA | As | Cyp51C | *Cladophialophora psammophila* CBS 110553 |
| XP_007754088.1 | C.yegresii_Cyp51B | Cyp51B | NA | As | Cyp51B | *Cladophialophora yegresii* CBS 114405 |
| XP_002614916.1 | C.lusitaniae_Cyp51 | Cyp51 | NA | As | Cyp51 | *Clavispora lusitaniae* ATCC 42720 |
| XP_001246802.1 | C.immitis_Cyp51A | Cyp51A | NA | As | Cyp51A | *Coccidioides immitis* RS |
| XP_001240306.1 | C.immitis_Cyp51B | Cyp51B | NA | As | Cyp51B | *Coccidioides immitis* RS |
| XP_003066446.1 | C.posadasii_Cyp51A | Cyp51A | NA | As | Cyp51A | *Coccidioides posadasii* C735 delta SOWgp |
| XP_003068142.1 | C.posadasii_Cyp51B | Cyp51B | NA | As | Cyp51B | *Coccidioides posadasii* C735 delta SOWgp |
| PIA15306.1 | C.reversa_Cyp51 | Cyp51 | Erg11 | Zo | Cyp51/Erg11^9^ | *Coemansia reversa* NRRL 1564 |
| XP_031884731.1 | C.fructicola_Cyp51A | Cyp51A | NA | As | Cyp51A | *Colletotrichum fructicola* |
| XP_031888323.1 | C.fructicola_Cyp51B | Cyp51B | NA | As | Cyp51B | *Colletotrichum fructicola* |
| XP_008090488.1 | C.graminicola_Cyp51B | Cyp51B | NA | As | Cyp51B | *Colletotrichum graminicola* M1.001 |
| XP_018163541.1 | C.higginsianum_Cyp51A | Cyp51A | Cyp51A | As | Cyp51A^8^ | *Colletotrichum higginsianum* IMI 349063 |
| XP_018163894.1 | C.higginsianum_Cyp51B | Cyp51B | NA | As | Cyp51B | *Colletotrichum higginsianum* IMI 349063 |
| XP_022474431.1 | C.orchidophilum_Cyp51A | Cyp51A | NA | As | Cyp51A | *Colletotrichum orchidophilum* |
| XP_022478445.1 | C.orchidophilum_Cyp51B | Cyp51B | NA | As | Cyp51B | *Colletotrichum orchidophilum* |
| XP_007765993.1 | C.puteana_Cyp51.1 | Cyp51 | NA | Ba | Cyp51 | *Coniophora puteana* RWD-64-598 SS2 |
| XP_007770297.1 | C.puteana_Cyp51.2 | Cyp51 | NA | Ba | Cyp51 | *Coniophora puteana* RWD-64-598 SS2 |
| XP_007781094.1 | C.apollinis_Cyp51B | Cyp51B | NA | As | Cyp51B | *Coniosporium apollinis* CBS 100218 |
| XP_001836522.1 | C.cinerea_Cyp51.1 | Cyp51 | NA | Ba | Cyp51 | *Coprinopsis cinerea* okayama7#130 |
| XP_001831760.1 | C.cinerea_Cyp51.2 | Cyp51 | NA | Ba | Cyp51 | *Coprinopsis cinerea* okayama7#130 |
| XP_018706205.1 | C.fumosorosea_Cyp51B | Cyp51B | NA | As | Cyp51B | *Cordyceps fumosorosea* ARSEF 2679 |
| XP_006668831.1 | C.militaris_Cyp51A | Cyp51A | Cyp51A | As | Cyp51A^8^ | *Cordyceps militaris* CM01 |
| XP_006670742.1 | C.militaris_Cyp51B | Cyp51B | NA | As | Cyp51B | *Cordyceps militaris* CM01 |
| XP_018994798.1 | C.amylolentus_Cyp51 | Cyp51 | NA | Ba | Cyp51 | *Cryptococcus amylolentus* CBS 6039 |
| XP_003191854.1 | C.gattii_Cyp51 | Cyp51 | NA | Ba | Cyp51 | *Cryptococcus gattii* WM276 |
| XP_012046482.1 | C.neoformans.H99_Cyp51 | Cyp51 | NA | Ba | Cyp51 | *Cryptococcus neoformans* var. grubii H99 |
| XP_566464.1 | C.neoformans.JEC21_Cyp51 | Cyp51 | NA | Ba | Cyp51 | *Cryptococcus neoformans* var. neoformans JEC21 |
| XP_019035794.1 | C.wingfieldii_Cyp51 | Cyp51 | NA | Ba | Cyp51 | *Cryptococcus wingfieldii* CBS 7118 |
| XP_018280711.1 | C.oleaginosum_Cyp51 | Cyp51 | NA | Ba | Cyp51 | *Cutaneotrichosporon oleaginosum* |
| XP_020073520.1 | C.jadinii_Cyp51 | Cyp51 | NA | As | Cyp51 | *Cyberlindnera jadinii* NRRL Y-1542 |
| XP_008719520.1 | C.europaea_Cyp51B | Cyp51B | NA | As | Cyp51B | *Cyphellophora europaea* CBS 101466 |
| XP_008721208.1 | C.europaea_Cyp51C | Cyp51C | NA | As | Cyp51C | *Cyphellophora europaea* CBS 101466 |
| EJU04979.1 | D.primogenitus_Cyp51 | Cyp51 | NA | Ba | Cyp51 | *Dacryopinax primogenitus* |
| XP_033437262.1 | D.childiae_Cyp51A.1 | Cyp51A | NA | As | Cyp51A | *Daldinia childiae* |
| XP_033439087.1 | D.childiae_Cyp51B | Cyp51B | NA | As | Cyp51B | *Daldinia childiae* |
| XP_015469227.1 | D.fabryi_Cyp51 | Cyp51 | NA | As | Cyp51 | *Debaryomyces fabryi* |
| XP_460143.1 | D.hansenii_Cyp51 | Cyp51 | NA | As | Cyp51 | *Debaryomyces hansenii* CBS767 |
| XP_007362342.1 | D.squalens_Cyp51.1 | Cyp51 | NA | Ba | Cyp51 | *Dichomitus squalens* LYAD-421 SS1 |
| XP_007366986.1 | D.squalens_Cyp51.2 | Cyp51 | NA | Ba | Cyp51 | *Dichomitus squalens* LYAD-421 SS1 |
| XP_033450066.1 | D.exigua_Cyp51B | Cyp51B | NA | As | Cyp51B | *Didymella exigua* CBS 183.55 |
| RKP33311.1 | D.cristalligena_Cyp51 | Cyp51 | NA | Zo | Cyp51 | *Dimargaris cristalligena* |
| XP_020129498.1 | D.corticola_Cyp51A | Cyp51A | NA | As | Cyp51A | *Diplodia corticola* |
| XP_020131421.1 | D.corticola_Cyp51B | Cyp51B | Cyp51B | As | Cyp51B^8^ | *Diplodia corticola* |
| XP_007785463.1 | E.pusillum_Cyp51A | Cyp51A | NA | As | Cyp51A | *Endocarpon pusillum* Z07020 |
| XP_007803001.1 | E.pusillum_Cyp51B | Cyp51B | NA | As | Cyp51B | *Endocarpon pusillum* Z07020 |
| XP_003647265.1 | E.cymbalariae_Cyp51 | Cyp51 | NA | As | Cyp51 | *Eremothecium cymbalariae* DBVPG#7215 |
| NP_984259.1 | E.gossypii_Cyp51 | Cyp51 | NA | As | Cyp51 | *Eremothecium gossypii* ATCC 10895 |
| XP_017987590.1 | E.sinecaudum_Cyp51 | Cyp51 | NA | As | Cyp51 | *Eremothecium sinecaudum* |
| XP_013255211.1 | E.aquamarina_Cyp51B | Cyp51B | NA | As | Cyp51B | *Exophiala aquamarina* CBS 119918 |
| XP_009152323.1 | E.dermatitidis_Cyp51B | Cyp51B | NA | As | Cyp51B | *Exophiala dermatitidis* NIH/UT8656 |
| XP_016226142.1 | E.mesophila_Cyp51B | Cyp51B | NA | As | Cyp51B | *Exophiala mesophila* |
| XP_016223848.1 | E.mesophila_Cyp51C | Cyp51C | NA | As | Cyp51C | *Exophiala mesophila* |
| XP_016262931.1 | E.oligosperma_Cyp51B | Cyp51B | NA | As | Cyp51B | *Exophiala oligosperma* |
| XP_016261164.1 | E.oligosperma_Cyp51C.1 | Cyp51C | NA | As | Cyp51C | *Exophiala oligosperma* |
| XP_016261165.1 | E.oligosperma_Cyp51C.2 | Cyp51C | NA | As | Cyp51C | *Exophiala oligosperma* |
| XP_016237740.1 | E.spinifera_Cyp51B | Cyp51B | NA | As | Cyp51B | *Exophiala spinifera* |
| XP_016231038.1 | E.spinifera_Cyp51C | Cyp51C | NA | As | Cyp51C | Exophiala spinifera |
| XP_013311440.1 | E.xenobiotica_Cyp51B | Cyp51B | NA | As | Cyp51B | *Exophiala xenobiotica* |
| XP_013320198.1 | E.xenobiotica_Cyp51C | Cyp51C | NA | As | Cyp51C | *Exophiala xenobiotica* |
| XP_008031238.1 | E.turcica_Cyp51B | Cyp51B | NA | As | Cyp51B | *Exserohilum turcica* Et28A |
| XP_012183538.1 | F.radiculosa_Cyp51 | Cyp51 | NA | Ba | Cyp51 | *Fibroporia radiculosa* |
| XP_007263187.1 | F.mediterranea_Cyp51 | Cyp51 | NA | Ba | Cyp51 | *Fomitiporia mediterranea* MF3/22 |
| XP_018689398.1 | F.erecta_Cyp51B | Cyp51B | NA | As | Cyp51B | *Fonsecaea erecta* |
| XP_018698687.1 | F.erecta_Cyp51C | Cyp51C | NA | As | Cyp51C | *Fonsecaea erecta* |
| XP_022507038.1 | F.monophora_Cyp51B | Cyp51B | NA | As | Cyp51B | *Fonsecaea monophora* |
| XP_022511803.1 | F.monophora_Cyp51C | Cyp51C | NA | As | Cyp51C | *Fonsecaea monophora* |
| XP_016631956.1 | F.multimorphosa_Cyp51B | Cyp51B | NA | As | Cyp51B | *Fonsecaea multimorphosa* CBS 102226 |
| XP_016637168.1 | F.multimorphosa_Cyp51C | Cyp51C | NA | As | Cyp51C | *Fonsecaea multimorphosa* CBS 102226 |
| XP_022494573.1 | F.nubica_Cyp51B | Cyp51B | NA | As | Cyp51B | *Fonsecaea nubica* |
| XP_022499888.1 | F.nubica_Cyp51C | Cyp51C | NA | As | Cyp51C | *Fonsecaea nubica* |
| XP_013286767.1 | F.pedrosoi_Cyp51B | Cyp51B | NA | As | Cyp51B | *Fonsecaea pedrosoi* CBS 271.37 |
| XP_013278994.1 | F.pedrosoi_Cyp51C | Cyp51C | NA | As | Cyp51C | *Fonsecaea pedrosoi* CBS 271.37 |
| XP_031010159.1 | F.coffeatum_Cyp51B | Cyp51B | NA | As | Cyp51B | *Fusarium coffeatum* |
| XP_031021383.1 | F.coffeatum_Cyp51C | Cyp51C | NA | As | Cyp51C | *Fusarium coffeatum* |
| XP_023435732.1 | F.fujikuroi_Cyp51A | Cyp51A | NA | As | Cyp51A | *Fusarium fujikuroi* IMI 58289 |
| XP_023424364.1 | F.fujikuroi_Cyp51B | Cyp51B | NA | As | Cyp51B | *Fusarium fujikuroi* IMI 58289 |
| XP_011321548.1 | F.graminearum_Cyp51A | Cyp51A | NA | As | Cyp51A | *Fusarium graminearum* PH-1 |
| XP_011316750.1 | F.graminearum_Cyp51B | Cyp51B | NA | As | Cyp51B | *Fusarium graminearum* PH-1 |
| XP_011325340.1 | F.graminearum_Cyp51C | Cyp51C | NA | As | Cyp51C | *Fusarium graminearum* PH-1 |
| XP_031067647.1 | F.oxysporum.cubense_Cyp51A | Cyp51A | NA | As | Cyp51A | *Fusarium oxysporum* f. sp. cubense tropical race 4 54006 |
| XP_031073139.1 | F.oxysporum.cubense_Cyp51B | Cyp51B | NA | As | Cyp51B | *Fusarium oxysporum* f. sp. cubense tropical race 4 54006 |
| XP_018232321.1 | F.oxysporum.lycopersici_Cyp51B | Cyp51B | NA | As | Cyp51B | *Fusarium oxysporum* f. sp. lycopersici 4287 |
| XP_018252291.1 | F.oxysporum.lycopersici_Cyp51C | Cyp51C | NA | As | Cyp51C | *Fusarium oxysporum* f. sp. lycopersici 4287 |
| XP_018249823.1 | F.oxysporum.lycopersici_Cyp51A | Cyp51A | NA | As | Cyp51A | *Fusarium oxysporum* f. sp. lycopersici 4287] |
| XP_031031054.1 | F.oxysporum.NRRL32931_Cyp51C | Cyp51C | NA | As | Cyp51C | *Fusarium oxysporum* NRRL 32931 |
| XP_031042981.1 | F.oxysporum.NRRL32932_Cyp51B | Cyp51B | NA | As | Cyp51B | *Fusarium oxysporum* NRRL 32931 |
| XP_031034287.1 | F.oxysporum.NRRL32933_Cyp51A | Cyp51A | NA | As | Cyp51A | *Fusarium oxysporum* NRRL 32931 |
| XP_031088172.1 | F.proliferatum_Cyp51A | Cyp51A | NA | As | Cyp51A | *Fusarium proliferatum* ET1 |
| XP_031075510.1 | F.proliferatum_Cyp51B | Cyp51B | NA | As | Cyp51B | *Fusarium proliferatum* ET1 |
| XP_031078374.1 | F.proliferatum_Cyp51C | Cyp51C | NA | As | Cyp51C | *Fusarium proliferatum* ET1 |
| XP_009251504.1 | F.pseudograminearum_Cyp51A | Cyp51A | NA | As | Cyp51A | *Fusarium pseudograminearum* CS3096 |
| XP_009252891.1 | F.pseudograminearum_Cyp51B | Cyp51B | NA | As | Cyp51B | *Fusarium pseudograminearum* CS3096 |
| XP_009253853.1 | F.pseudograminearum_Cyp51C | Cyp51C | NA | As | Cyp51C | *Fusarium pseudograminearum* CS3096 |
| XP_025586427.1 | F.venenatum_Cyp51A | Cyp51A | NA | As | Cyp51A | *Fusarium venenatum* |
| XP_025588470.1 | F.venenatum_Cyp51B | Cyp51B | NA | As | Cyp51B | *Fusarium venenatum* |
| XP_025594088.1 | F.venenatum_Cyp51C | Cyp51C | NA | As | Cyp51C | *Fusarium venenatum* |
| XP_018757407.1 | F.verticillioides_Cyp51A | Cyp51A | NA | As | Cyp51A | *Fusarium verticillioides* 7600 |
| XP_018743733.1 | F.verticillioides_Cyp51B | Cyp51B | NA | As | Cyp51B | *Fusarium verticillioides* 7600 |
| XP_018760287.1 | F.verticillioides_Cyp51C | Cyp51C | NA | As | Cyp51C | *Fusarium verticillioides* 7600 |
| XP_009226432.1 | G.tritici_Cyp51B | Cyp51B | NA | As | Cyp51B | *Gaeumannomyces tritici* R3-111a-1 |
| XP_008080642.1 | G.lozoyensis_Cyp51B | Cyp51B | NA | As | Cyp51B | *Glarea lozoyensis* ATCC 20868 |
| XP_007867646.1 | G.trabeum_Cyp51 | Cyp51 | NA | Ba | Cyp51 | *Gloeophyllum trabeum* ATCC 11539 |
| KXS21615.1 | G.prolifera_Cyp51 | Cyp51 | NA | Mo | Cyp51 | *Gonapodya prolifera* JEL478 |
| XP_014171966.1 | G.clavigera_Cyp51B | Cyp51B | NA | As | Cyp51B | *Grosmannia clavigera* kw1407 |
| XP_009544647.1 | H.irregulare_Cyp51 | Cyp51 | NA | Ba | Cyp51 | *Heterobasidion irregulare* TC 32-1 |
| XP_001540641.1 | H.capsulatum_Cyp51A | Cyp51A | NA | As | Cyp51A | *Histoplasma capsulatum* NAm1 |
| XP_001540208.1 | H.capsulatum_Cyp51B | Cyp51B | NA | As | Cyp51B | *Histoplasma capsulatum* NAm1 |
| XP_024739256.1 | H.bicolor_Cyp51B.1 | Cyp51B | Cyp51B | As | Cyp51B^8^ | *Hyaloscypha bicolor* E |
| XP_024728738.1 | H.bicolor_Cyp51B.2 | Cyp51B | NA | As | Cyp51B | *Hyaloscypha bicolor* E |
| XP_020074335.1 | H.burtonii_Cyp51.1 | Cyp51 | NA | As | Cyp51 | *Hyphopichia burtonii* NRRL Y-1933 |
| XP_020074334.1 | H.burtonii_Cyp51.2 | Cyp51 | NA | As | Cyp51 | *Hyphopichia burtonii* NRRL Y-1933 |
| XP_025359545.1 | J.rosea_Cyp51 | Cyp51 | NA | Ba | Cyp51 | *Jaminaea rosea* |
| XP_016291895.1 | K.brasiliensis_Cyp51 | Cyp51 | NA | Ba | Cyp51 | *Kalmanozyma brasiliensis* GHG001 |
| XP_003956698.1 | K.africana_Cyp51 | Cyp51 | NA | As | Cyp51 | *Kazachstania africana* CBS 2517 |
| XP_022463022.1 | K.naganishii_Cyp51 | Cyp51 | NA | As | Cyp51 | *Kazachstania naganishii* CBS 8797 |
| XP_454109.1 | K.lactis_Cyp51 | Cyp51 | NA | As | Cyp51 | *Kluyveromyces lactis* |
| XP_022674874.1 | K.marxianus_Cyp51 | Cyp51 | NA | As | Cyp51 | *Kluyveromyces marxianus* DMKU3-1042 |
| XP_021870957.1 | K.imperatae_Cyp51 | Cyp51 | NA | Ba | Cyp51 | *Kockovaella imperatae* |
| XP_002493197.1 | K.phaffii_Cyp51 | Cyp51 | NA | As | Cyp51 | *Komagataella phaffii* GS115 |
| XP_022461631.1 | K.capsulata_Cyp51 | Cyp51 | NA | As | Cyp51 | *Kuraishia capsulata* CBS 1993 |
| XP_019046080.1 | K.bestiolae_Cyp51 | Cyp51 | NA | Ba | Cyp51 | *Kwoniella bestiolae* CBS 10118 |
| XP_018263285.1 | K.dejecticola_Cyp51 | Cyp51 | NA | Ba | Cyp51 | *Kwoniella dejecticola* CBS 10117 |
| XP_019007085.1 | K.mangroviensis_Cyp51 | Cyp51 | NA | Ba | Cyp51 | *Kwoniella mangroviensis* CBS 8507 |
| XP_019010751.1 | K.pini_Cyp51 | Cyp51 | NA | Ba | Cyp51 | *Kwoniella pini* CBS 10737 |
| XP_001880615.1 | L.bicolor_Cyp51 | Cyp51 | NA | Ba | Cyp51 | *Laccaria bicolor* S238N-H82 |
| XP_022629925.1 | L.lanzarotensis_Cyp51 | Cyp51 | NA | As | Cyp51 | *Lachancea lanzarotensis* |
| XP_002555262.1 | L.thermotoleran_Cyp51 | Cyp51 | NA | As | Cyp51 | *Lachancea thermotolerans* CBS 6340 |
| XP_031003078.1 | L.hyalina_Cyp51B | Cyp51B | NA | As | Cyp51B | *Lachnellula hyalina* |
| SLM33450.1 | L.pustulata_Cyp51B.1 | Cyp51B | Cyp51B | As | Cyp51B^8^ | *Lasallia pustulata* |
| KAA6415496.1 | L.pustulata_Cyp51B.2 | Cyp51B | NA | As | Cyp51B | *Lasallia pustulata* |
| XP_003840561.1 | L.maculans_Cyp51B | Cyp51B | NA | As | Cyp51B | *Leptosphaeria maculans* JN3 |
| ORX72854.1 | L.pennispora_Cyp51 | Cyp51 | NA | Zo | Cyp51 | *Linderina pennispora* |
| XP_021876902.1 | L.transversale_Cyp51 | Cyp51 | NA | Mu | Cyp51 | *Lobosporangium transversale* |
| XP_001524706.1 | L.elongisporus_Cyp51 | Cyp51 | NA | As | Cyp51 | *Lodderomyces elongisporus* NRRL YB-4239 |
| XP_001730619.1 | M.globosa_Cyp51 | Cyp51 | NA | Ba | Cyp51 | *Malassezia globosa* CBS 7966 |
| XP_027485371.1 | M.restricta_Cyp51 | Cyp51 | NA | Ba | Cyp51 | *Malassezia restricta* |
| XP_018740314.1 | M.sympodialis_Cyp51 | Cyp51 | NA | Ba | Cyp51 | *Malassezia sympodialis* ATCC 42132 |
| XP_007290460.1 | M.brunnea_Cyp51B | Cyp51B | NA | As | Cyp51B | *Marssonina brunnea* f. sp. 'multigermtubi' MB_m1 |
| XP_025357709.1 | M.miltonrushii_Cyp51 | Cyp51 | NA | Ba | Cyp51 | *Meira miltonrushii* |
| XP_007418520.1 | M.laricipopulina_Cyp51 | Cyp51 | NA | Ba | Cyp51 | *Melampsora larici-populina* 98AG31 |
| XP_007813443.1 | M.acridum_Cyp51B | Cyp51B | NA | As | Cyp51B | *Metarhizium acridum* CQMa 102 |
| XP_014540248.1 | M.brunneum_Cyp51A | Cyp51A | Cyp51B | As | Cyp51A^9^ | *Metarhizium brunneum* ARSEF 3297 |
| XP_014546348.1 | M.brunneum_Cyp51B | Cyp51B | Cyp51B | As | Cyp51B^8^ | *Metarhizium brunneum* ARSEF 3297 |
| XP_007817531.2 | M.robertsii_Cyp51A | Cyp51A | Cyp51F2 | As | Cyp51A^9^ | *Metarhizium robertsii* ARSEF 23 |
| XP_007820238.1 | M.robertsii_Cyp51B | Cyp51B | Cyp51F1 | As | Cyp51B^9^ | *Metarhizium robertsii* ARSEF 23 |
| XP_001484034.1 | M.guilliermondii_Cyp51 | Cyp51 | NA | As | Cyp51 | *Meyerozyma guilliermondii* ATCC 6260 |
| XP_002845046.1 | M.canis_Cyp51A | Cyp51A | NA | As | Cyp51A | *Microsporum canis* CBS 113480 |
| XP_002843524.1 | M.canis_Cyp51B | Cyp51B | NA | As | Cyp51B | *Microsporum canis* CBS 113480 |
| XP_014569710.1 | M.osmundae_Cyp51 | Cyp51 | NA | Ba | Cyp51 | *Mixia osmundae* IAM 14324 |
| XP_014655647.1 | M.antarcticus_Cyp51 | Cyp51 | NA | Ba | Cyp51 | *Moesziomyces antarcticus* |
| XP_003169749.1 | N.gypsea_Cyp51A | Cyp51A | NA | As | Cyp51A | *Nannizzia gypsea* CBS 118893 |
| XP_003172213.1 | N.gypsea_Cyp51B | Cyp51B | YjeF | As | Cyp51B^9^ | *Nannizzia gypsea* CBS 118893 |
| XP_003677336.1 | N.castellii_Cyp51 | Cyp51 | NA | As | Cyp51 | *Naumovozyma castellii* CBS 4309 |
| XP_003670672.1 | N.dairenensis_Cyp51 | Cyp51 | NA | As | Cyp51 | *Naumovozyma dairenensis* CBS 421 |
| XP_003045204.1 | N.haematococca_Cyp51A | Cyp51A | NA | As | Cyp51A | *Nectria haematococca* mpVI 77-13-4 |
| XP_003054236.1 | N.haematococca_Cyp51B | Cyp51B | NA | As | Cyp51B | *Nectria haematococca* mpVI 77-13-4 |
| XP_003051421.1 | N.haematococca_Cyp51C | Cyp51C | NA | As | Cyp51C | *Nectria haematococca* mpVI 77-13-4 |
| XP_964049.2 | N.crassa_Cyp51B | Cyp51B | NA | As | Cyp51B | *Neurospora crassa* OR74A |
| XP_009856659.1 | N.tetrasperma_Cyp51B | Cyp51B | NA | As | Cyp51B | *Neurospora tetrasperma* FGSC 2508 |
| XP_013934130.1 | O.parapolymorpha_Cyp51 | Cyp51 | NA | As | Cyp51 | *Ogataea parapolymorpha* DL-1 |
| XP_018211714.1 | O.polymorpha_Cyp51 | Cyp51 | NA | As | Cyp51 | *Ogataea polymorpha* |
| XP_010759277.1 | P.brasiliensis_Cyp51B | Cyp51B | NA | As | Cyp51B | *Paracoccidioides brasiliensis* Pb18 |
| XP_002796968.2 | P.lutzii_Cyp51B | Cyp51B | NA | As | Cyp51B | *Paracoccidioides lutzii* Pb01 |
| XP_018030695.1 | P.sporulosa_Cyp51B | Cyp51B | NA | As | Cyp51B | *Paraphaeosphaeria sporulosa* |
| XP_001794253.1 | P.nodorum_Cyp51B | Cyp51B | NA | As | Cyp51B | *Parastagonospora nodorum* SN15 |
| XP_022577516.1 | P.zonata_Cyp51B.2 | Cyp51B | NA | As | Cyp51B | *Penicilliopsis zonata* CBS 506.65 |
| XP_022484282.1 | P.arizonense_Cyp51A | Cyp51A | NA | As | Cyp51A | *Penicillium arizonense* |
| XP_022489849.1 | P.arizonense_Cyp51B | Cyp51B | NA | As | Cyp51B | *Penicillium arizonense* |
| XP_014532172.1 | P.digitatum_Cyp51A | Cyp51A | Cyp51A | As | Cyp51A^8^ | *Penicillium digitatum* Pd1 |
| XP_014538931.1 | P.digitatum_Cyp51B | Cyp51B | NA | As | Cyp51B | *Penicillium digitatum* Pd1 |
| XP_016598797.1 | P.expansum_Cyp51A | Cyp51A | NA | As | Cyp51A | *Penicillium expansum* |
| XP_016599458.1 | P.expansum_Cyp51B | Cyp51B | NA | As | Cyp51B | *Penicillium expansum* |
| XP_002557907.1 | P.rubens_Cyp51A | Cyp51A | NA | As | Cyp51A | *Penicillium rubens* Wisconsin 54-1255 |
| XP_007830361.1 | P.fici_Cyp51A | Cyp51A | NA | As | Cyp51A | *Pestalotiopsis fici* W106-1 |
| XP_007828197.1 | P.fici_Cyp51B | Cyp51B | NA | As | Cyp51B | *Pestalotiopsis fici* W106-1 |
| XP_007919391.1 | P.minimum_Cyp51A | Cyp51A | NA | As | Cyp51A | *Phaeoacremonium minimum* UCRPA7 |
| XP_007913161.1 | P.minimum_Cyp51B | Cyp51B | NA | As | Cyp51B | *Phaeoacremonium minimum* UCRPA7 |
| XP_007918697.1 | P.minimum_Cyp51C | Cyp51C | NA | As | Cyp51C | *Phaeoacremonium minimum* UCRPA7 |
| XP_007403144.1 | P.carnosa_Cyp51 | Cyp51 | NA | Ba | Cyp51 | *Phanerochaete carnosa* HHB-10118-sp |
| XP_030995983.1 | P.curvata_Cyp51B | Cyp51B | NA | As | Cyp51B | *Phialemoniopsis curvata* |
| XP_030998229.1 | P.curvata_Cyp51C | Cyp51C | NA | As | Cyp51C | *Phialemoniopsis curvata* |
| XP_018076846.1 | P.scopiformis_Cyp51B | Cyp51B | NA | As | Cyp51B | *Phialocephala scopiformis* |
| XP_018005711.1 | P.attinorum_Cyp51B | Cyp51B | NA | As | Cyp51B | *Phialophora attinorum* |
| XP_018002049.1 | P.attinorum_Cyp51C | Cyp51C | NA | As | Cyp51C | *Phialophora attinorum* |
| XP_018297712.1 | P.blakesleeanus_Cyp51 | Cyp51 | NA | Mu | Cyp51 | *Phycomyces blakesleeanus* NRRL 1555(-) |
| XP_029322955.1 | P.kudriavzevii_Cyp51 | Cyp51 | NA | As | Cyp51 | *Pichia kudriavzevii* |
| XP_019020668.1 | P.membranifaciens_Cyp51 | Cyp51 | NA | As | Cyp51 | *Pichia membranifaciens* NRRL Y-2026 |
| XP_018226934.1 | P.carinii_Cyp51 | Cyp51 | NA | As | Cyp51 | *Pneumocystis carinii* B80 |
| XP_007872980.1 | P.murina_Cyp51 | Cyp51 | NA | As | Cyp51 | *Pneumocystis murina* B123 |
| XP_022285666.1 | P.chlamydosporia_Cyp51A | Cyp51A | NA | As | Cyp51A | *Pochonia chlamydosporia* 170 |
| XP_018148691.1 | P.chlamydosporia_Cyp51B | Cyp51B | NA | As | Cyp51B | *Pochonia chlamydosporia* 170 |
| XP_001912649.1 | P.anserina_Cyp51B | Cyp51B | NA | As | Cyp51B | *Podospora anserina* S mat+ |
| XP_024335677.1 | P.placenta_Cyp51 | Cyp51 | NA | Ba | Cyp51 | *Postia placenta* MAD-698-R-SB12 |
| XP_007928752.1 | P.fijiensis_Cyp51B | Cyp51B | NA | As | Cyp51B | *Pseudocercospora fijiensis* CIRAD86 |
| XP_024322169.1 | P.destructans_Cyp51B | Cyp51B | NA | As | Cyp51B | *Pseudogymnoascus destructans* |
| XP_018135480.1 | P.verrucosus_Cyp51B | Cyp51B | NA | As | Cyp51B | *Pseudogymnoascus verrucosus* |
| XP_025349417.1 | P.glucosiphilum_Cyp51 | Cyp51 | NA | Ba | Cyp51 | Pseudomicrostroma glucosiphilum |
| XP_012192750.1 | P.hubeiensis_Cyp51 | Cyp51 | NA | Ba | Cyp51 | *Pseudozyma hubeiensis* SY62 |
| XP_007388976.1 | P.strigosozonata_Cyp51 | Cyp51 | NA | Ba | Cyp51 | *Punctularia strigosozonata* HHB-11173 SS5 |
| XP_018180143.1 | P.lilacinum_Cyp51A | Cyp51A | NA | As | Cyp51A | *Purpureocillium lilacinum* |
| XP_018180650.1 | P.lilacinum_Cyp51B | Cyp51B | NA | As | Cyp51B | *Purpureocillium lilacinum* |
| XP_001939762.1 | P.triticirepentis_Cyp51A | Cyp51A | NA | As | Cyp51A | *Pyrenophora tritici-repentis* Pt-1C-BFP |
| XP_001939023.1 | P.triticirepentis_Cyp51B | Cyp51B | NA | As | Cyp51B | *Pyrenophora tritici-repentis* Pt-1C-BFP |
| XP_030984970.1 | P.grisea_Cyp51A | Cyp51A | NA | As | Cyp51A | *Pyricularia grisea* |
| XP_030984665.1 | P.grisea_Cyp51B | Cyp51B | NA | As | Cyp51B | *Pyricularia grisea* |
| XP_003710875.1 | P.oryzae_Cyp51A | Cyp51A | NA | As | Cyp51A | *Pyricularia oryzae* 70-15 |
| XP_003713527.1 | P.oryzae_Cyp51B | Cyp51B | NA | As | Cyp51B | *Pyricularia oryzae* 70-15 |
| XP_029751269.1 | P.pennisetigena_Cyp51B | Cyp51B | NA | As | Cyp51B | *Pyricularia pennisetigena* |
| XP_023625135.1 | R.collocygni_Cyp51B | Cyp51B | NA | As | Cyp51B | *Ramularia collo-cygni* |
| XP_013327734.1 | R.emersonii_Cyp51B | Cyp51B | NA | As | Cyp51B | *Rasamsonia emersonii* CBS 393.64 |
| XP_013267469.1 | R.mackenziei_Cyp51B | Cyp51B | NA | As | Cyp51B | *Rhinocladiella mackenziei* CBS 650.93 |
| XP_025190100.1 | R.irregularis_Cyp51 | Cyp51 | NA | Mu | Cyp51 | *Rhizophagus irregularis* DAOM 181602=DAOM 197198 |
| XP_023464051.1 | R.microsporus_Cyp51.1 | Cyp51 | NA | Mu | Cyp51 | *Rhizopus microsporus* ATCC 52813 |
| XP_023471226.1 | R.microsporus_Cyp51.2 | Cyp51 | NA | Mu | Cyp51 | *Rhizopus microsporus* ATCC 52813 |
| XP_016271186.1 | R.toruloides_Cyp51 | Cyp51 | NA | Ba | Cyp51 | *Rhodotorula toruloides* NP11 |
| NP_011871.1 | S.cerevisiae_Cyp51 | Cyp51 | NA | As | Cyp51 | *Saccharomyces cerevisiae* S288C |
| XP_018219245.1 | S.eubayanus_Cyp51 | Cyp51 | NA | As | Cyp51 | *Saccharomyces eubayanus* |
| XP_019022946.1 | S.complicata_Cyp51 | Cyp51 | NA | As | Cyp51 | *Saitoella complicata* NRRL Y-17804 |
| XP_031852458.1 | S.ingens_Cyp51 | Cyp51 | NA | As | Cyp51 | *Saprochaete ingens* |
| XP_016644011.1 | S.apiospermum_Cyp51B | Cyp51B | NA | As | Cyp51B | *Scedosporium apiospermum* |
| XP_001386140.2 | S.stipitis_Cyp51 | Cyp51 | CypLI | As | Cyp51^9^ | *Scheffersomyces stipitis* CBS 6054 |
| XP_003031999.1 | S.commune_Cyp51 | Cyp51 | NA | Ba | Cyp51 | *Schizophyllum commune* H4-8 |
| XP_013021772.1 | S.cryophilus_Cyp51 | Cyp51 | NA | As | Cyp51 | *Schizosaccharomyces cryophilus* OY26 |
| XP_002174883.2 | S.japonicus_Cyp51 | Cyp51 | NA | As | Cyp51 | *Schizosaccharomyces japonicus* yFS275 |
| XP_013015904.1 | S.octosporus_Cyp51 | Cyp51 | NA | As | Cyp51 | *Schizosaccharomyces octosporus* yFS286 |
| NP_592990.1 | S.pombe_Cyp51 | Cyp51 | NA | As | Cyp51 | *Schizosaccharomyces pombe* |
| XP_001594997.1 | S.sclerotiorum_Cyp51B | Cyp51B | NA | As | Cyp51B | *Sclerotinia sclerotiorum* 1980 UF-70 |
| XP_007318458.1 | S.lacrymans_Cyp51 | Cyp51 | NA | Ba | Cyp51 | *Serpula lacrymans* var. lacrymans S7.9 |
| PWA03462.1 | S.angustum_Cyp51 | Cyp51 | NA | Zo | Cyp51 | *Smittium angustum* |
| OMJ14618.1 | S.culicis_Cyp51.1 | Cyp51 | NA | Zo | Cyp51 | *Smittium culicis* |
| OMJ17766.1 | S.culicis_Cyp51.2 | Cyp51 | NA | Zo | Cyp51 | *Smittium culicis* |
| PVV01034.1 | S.megazygosporum_Cyp51 | Cyp51 | NA | Zo | Cyp51 | *Smittium megazygosporum* |
| OLY85446.1 | S.mucronatum_Cyp51 | Cyp51 | NA | Zo | Cyp51 | *Smittium mucronatum* |
| PVU92872.1 | S.simulii_Cyp51 | Cyp51 | NA | Zo | Cyp51 | *Smittium simulii* |
| XP_028467074.1 | S.alkalinus_Cyp51A | Cyp51A | NA | As | Cyp51A | *Sodiomyces alkalinus* F11 |
| XP_028465183.1 | S.alkalinus_Cyp51B | Cyp51B | NA | As | Cyp51B | *Sodiomyces alkalinus* F11 |
| XP_003346200.1 | S.macrospora_Cyp51B | Cyp51B | NA | As | Cyp51B | *Sordaria macrospora k-hell* |
| XP_016758426.1 | S.musiva_Cyp51B | Cyp51B | NA | As | Cyp51B | *Sphaerulina musiva* SO2202 |
| XP_016612382.1 | S.punctatus_Cyp51 | Cyp51 | NA | Ch | Cyp51 | *Spizellomyces punctatus* DAOM BR117 |
| XP_029741792.1 | S.graminicola_Cyp51 | Cyp51 | NA | Ba | Cyp51 | *Sporisorium graminicola* |
| XP_016587088.1 | S.schenckii_Cyp51B | Cyp51B | NA | As | Cyp51B | *Sporothrix schenckii* 1099-18 |
| XP_007300062.1 | S.hirsutum_Cyp51.1 | Cyp51 | NA | Ba | Cyp51 | *Stereum hirsutum* FP-91666 SS1 |
| XP_007305230.1 | S.hirsutum_Cyp51.2 | Cyp51 | NA | Ba | Cyp51 | *Stereum hirsutum* FP-91666 SS1 |
| XP_018737268.1 | S.lignohabitans_Cyp51 | Cyp51 | NA | As | Cyp51 | *Sugiyamaella lignohabitans* |
| XP_020062236.1 | S.tanzawaensis_Cyp51 | Cyp51 | NA | As | Cyp51 | *Suhomyces tanzawaensis* NRRL Y-17324 |
| RKP27255.1 | S.pseudoplumigaleata_Cyp51 | Cyp51 | NA | Zo | Cyp51 | *Syncephalis pseudoplumigaleata* |
| XP_031025773.1 | S.microbalum_Cyp51 | Cyp51 | NA | Ch | Cyp51 | *Synchytrium microbalum* |
| XP_020121104.1 | T.atroroseus_Cyp51B | Cyp51B | NA | As | Cyp51B | *Talaromyces atroroseus* |
| XP_002146405.1 | T.marneffei_Cyp51B | Cyp51B | Cyp51B | As | Cyp51B^8^ | *Talaromyces marneffei* ATCC 18224 |
| XP_002480262.1 | T.stipitatus_Cyp51B.1 | Cyp51B | NA | As | Cyp51B | *Talaromyces stipitatus* ATCC 10500 |
| XP_002478695.1 | T.stipitatus_Cyp51B.2 | Cyp51B | Cyp51B | As | Cyp51B^8^ | *Talaromyces stipitatus* ATCC 10500 |
| XP_004180856.1 | T.blattae_Cyp51 | Cyp51 | NA | As | Cyp51 | *Tetrapisispora blattae* CBS 6284 |
| XP_003685341.1 | T.phaffii_Cyp51 | Cyp51 | NA | As | Cyp51 | *Tetrapisispora phaffii* CBS 4417 |
| XP_003658636.1 | T.thermophilus_Cyp51B | Cyp51B | NA | As | Cyp51B | *Thermothelomyces thermophilus* ATCC 42464 |
| XP_003656603.1 | T.terrestris_Cyp51B | Cyp51B | NA | As | Cyp51B | *Thermothielavioides terrestris* NRRL 8126 |
| XP_013241633.1 | T.anomala_Cyp51 | Cyp51 | NA | Ba | Cyp51 | *Tilletiaria anomala* UBC 951 |
| XP_003678998.1 | T.delbrueckii_Cyp51 | Cyp51 | NA | As | Cyp51 | *Torulaspora delbrueckii* |
| XP_008039623.1 | T.versicolor_Cyp51 | Cyp51 | NZ | BA | Cyp51 | *Trametes versicolor FP-101664 SS1* |
| XP_007003323.1 | T.mesenterica_Cyp51 | Cyp51 | NA | Ba | Cyp51 | *Tremella mesenterica* DSM 1558 |
| XP_024759275.1 | T.asperellum_Cyp51B | Cyp51B | NA | As | Cyp51B | *Trichoderma asperellum* CBS 433.97 |
| XP_013948124.1 | T.atroviride_Cyp51B | Cyp51B | NA | As | Cyp51B | *Trichoderma atroviride* IMI 206040 |
| XP_013937523.1 | T.atroviride_Cyp51A | Cyp51A | NA | As | Cyp51A | *Trichoderma atroviride* IMI 206040] |
| XP_024750384.1 | T.citrinoviride_Cyp51B | Cyp51B | NA | As | Cyp51B | *Trichoderma citrinoviride* |
| XP_018662524.1 | T.gamsii_Cyp51A | Cyp51A | NA | As | Cyp51A | *Trichoderma gamsii* |
| XP_018663075.1 | T.gamsii_Cyp51B | Cyp51B | NA | As | Cyp51B | *Trichoderma gamsii* |
| XP_024768437.1 | T.harzianum_Cyp51A | Cyp51A | NA | As | Cyp51A | *Trichoderma harzianum* CBS 226.95 |
| XP_024773840.1 | T.harzianum_Cyp51B | Cyp51B | NA | As | Cyp51B | *Trichoderma harzianum* CBS 226.95 |
| XP_006962756.1 | T.reesei_Cyp51B | Cyp51B | NA | As | Cyp51B | *Trichoderma reesei* QM6a |
| XP_013951699.1 | T.virens_Cyp51B | Cyp51B | NA | As | Cyp51B | *Trichoderma virens* Gv29-8 |
| XP_003012699.1 | T.benhamiae_Cyp51B | Cyp51B | NA | As | Cyp51B | *Trichophyton benhamiae* CBS 112371 |
| XP_003235929.1 | T.rubrum_Cyp51A | Cyp51A | NA | As | Cyp51A | *Trichophyton rubrum* CBS 118892 |
| XP_003236980.1 | T.rubrum_Cyp51B | Cyp51B | NA | As | Cyp51B | *Trichophyton rubrum* CBS 118892 |
| XP_003025076.1 | T.verrucosum_Cyp51B.2 | Cyp51B | NA | As | Cyp51B | *Trichophyton verrucosum* HKI 0517 |
| XP_014184103.1 | T.asahii_Cyp51 | Cyp51 | NA | Ba | Cyp51 | *Trichosporon asahii* var. asahii CBS 2479 |
| XP_011390148.1 | U.maydis_Cyp51 | Cyp51 | NA | Ba | Cyp51 | *Ustilago maydis* 521 |
| XP_001646750.1 | V.polyspora_Cyp51.1 | Cyp51 | NA | As | Cyp51 | *Vanderwaltozyma polyspora* DSM 70294 |
| XP_001645542.1 | V.polyspora_Cyp51.2 | Cyp51 | NA | As | Cyp51 | *Vanderwaltozyma polyspora* DSM 70294 |
| XP_016211553.1 | V.gallopava_Cyp51B | Cyp51B | NA | As | Cyp51B | *Verruconis gallopava* |
| XP_028497475.1 | V.nonalfalfae_Cyp51B | Cyp51B | NA | As | Cyp51B | *Verticillium nonalfalfae* |
| XP_009269638.1 | W.ichthyophaga_Cyp51 | Cyp51 | NA | Ba | Cyp51 | *Wallemia ichthyophaga* EXF-994 |
| XP_006959970.1 | W.mellicola_Cyp51.1 | Cyp51 | NA | Ba | Cyp51 | *Wallemia mellicola* CBS 633.66 |
| XP_006955920.1 | W.mellicola_Cyp51.2 | Cyp51 | NA | Ba | Cyp51 | *Wallemia mellicola* CBS 633.66 |
| XP_024662554.1 | W.sorbophila_Cyp51 | Cyp51 | NA | As | Cyp51 | *Wickerhamiella sorbophila* |
| XP_019037906.1 | W.anomalus_Cyp51 | Cyp51 | NA | As | Cyp51 | *Wickerhamomyces anomalus* NRRL Y-366-8 |
| XP_011271347.1 | W.ciferrii_Cyp51 | Cyp51 | NA | As | Cyp51 | *Wickerhamomyces ciferrii* |
| XP_018189130.1 | X.heveae_Cyp51A | Cyp51A | Cyp51A | As | Cyp51A^8^ | *Xylona heveae* TC161 |
| XP_018192447.1 | X.heveae_Cyp51B | Cyp51B | NA | As | Cyp51B | *Xylona heveae* TC161 |
| XP_006687406.1 | Y.tenuis_Cyp51 | Cyp51 | NA | As | Cyp51 | *Yamadazyma tenuis* ATCC 10573 |
| XP_500518.1 | Y.lipolytica_Cyp51 | Cyp51 | NA | As | Cyp51 | *Yarrowia lipolytica* CLIB122 |
| OMH83707.1 | Z.culisetae_Cyp51 | Cyp51 | Erg11 | Zo | Cyp51/Erg11^9^ | *Zancudomyces culisetae* |
| XP_002497042.1 | Z.rouxii_Cyp51 | Cyp51 | NA | As | Cyp51 | *Zygosaccharomyces rouxii* |
| XP_003851092.1 | Z.tritici_Cyp51B | Cyp51B | Erg11 | As | Cyp51B^9^ | *Zymoseptoria tritici* IPO323 |

^1^Accession number in NCBI (https://www.ncbi.nlm.nih.gov/)

^2^Based on species name and clade designation from Supplemental Figure 1

^3^Based on clade designation from Supplemental Figure 1

^4^Current name in NCBI (https://www.ncbi.nlm.nih.gov/)

^5^Phyla based on NCBI (<https://www.ncbi.nlm.nih.gov/>)

^6^Proposed name for proteins with no current name in NCBI (https://www.ncbi.nlm.nih.gov/), based on clade designation from Supplemental Figure 1

^7^Species and strain based on NCBI (<https://www.ncbi.nlm.nih.gov/>)

^8^Proposed name is the same as current name in NCBI

^9^Proposed name is different from current name in NCBI
