# Supplementary material for: Analysis of Cyp51 protein sequences shows 4 major Cyp51 gene family groups across fungi
Source: G3 (Bethesda). 2022 Sep 21;12(11):jkac249. doi: 10.1093/g3journal/jkac249 (PMC9635630; doi:10.1093/g3journal/jkac249)
Supplement: jkac249_Supplemental_Table_S3 [file jkac249_supplemental_table_s3.docx]

**Supplemental Table 3. Cyp51 Protein Similarity Across 4 Cyp51 Groups**

| Similarity between all Cyp51 protein sequences in each group^1^ | | | | |
| --- | --- | --- | --- | --- |
| Whole Protein | Cyp51 | Cyp51A | Cyp51B | Cyp51C |
| Cyp51 | 45.6% |  |  |  |
| Cyp51A | 46.9% | 68.8% |  |  |
| Cyp51B | 49.5% | 60.5% | 64.7% |  |
| Cyp51C | 44.6% | 59.4% | 60.8% | 67.0% |

^1^Similarity is based on Geneious Prime’s pairwise identity function.
