# Supplementary material for: Analysis of Cyp51 protein sequences shows 4 major Cyp51 gene family groups across fungi
Source: G3 (Bethesda). 2022 Sep 21;12(11):jkac249. doi: 10.1093/g3journal/jkac249 (PMC9635630; doi:10.1093/g3journal/jkac249)
Supplement: jkac249_Supplemental_Table_S4 [file jkac249_supplemental_table_s4.docx]

**Supplemental Table 4. Similarity of Cyp51 motifs between groups.**

| Similarity between consensus sequences from each group^1,2^ | | | | | Similarity between all sequences from each group^2^ | | | | |
| --- | --- | --- | --- | --- | --- | --- | --- | --- | --- |
| SRS1 | Cyp51 | Cyp51A | Cyp51B | Cyp51C | SRS1 | Cyp51 | Cyp51A | Cyp51B | Cyp51C |
| Cyp51 | 100% |  |  |  | Cyp51 | 78% |  |  |  |
| Cyp51A | 78.95% | 100% |  |  | Cyp51A | 76.9% | 89.8% |  |  |
| Cyp51B | 78.95% | 94.74% | 100% |  | Cyp51B | 78.6% | 84.7% | 85.2% |  |
| Cyp51C | 68.42% | 68.42% | 73.68% | 100% | Cyp51C | 75.4% | 81.8% | 81.5% | 80.5% |
| SRS2 | Cyp51 | Cyp51A | Cyp51B | Cyp51C | SRS2 | Cyp51 | Cyp51A | Cyp51B | Cyp51C |
| Cyp51 | 100% |  |  |  | Cyp51 | 69.7% |  |  |  |
| Cyp51A | 100% | 100% |  |  | Cyp51A | 73.0% | 84.2% |  |  |
| Cyp51B | 85.71% | 85.71% | 100% |  | Cyp51B | 77.5% | 87.5% | 93.7% |  |
| Cyp51C | 42.86% | 42.86% | 42.86% | 100% | Cyp51C | 65.3% | 72.8% | 83.8% | 91.5% |
| SRS3 | Cyp51 | Cyp51A | Cyp51B | Cyp51C | SRS3 | Cyp51 | Cyp51A | Cyp51B | Cyp51C |
| Cyp51 | 100% |  |  |  | Cyp51 | 49.9% |  |  |  |
| Cyp51A | 45.45% | 100% |  |  | Cyp51A | 49.2% | 72.5% |  |  |
| Cyp51B | 63.64% | 72.73% | 100% |  | Cyp51B | 54.0% | 63.1% | 68.3% |  |
| Cyp51C | 54.55% | 45.45% | 54.55% | 100% | Cyp51C | 48.4% | 61.0% | 64.2% | 67.1% |
| SRS4 | Cyp51 | Cyp51A | Cyp51B | Cyp51C | SRS4 | Cyp51 | Cyp51A | Cyp51B | Cyp51C |
| Cyp51 | 100% |  |  |  | Cyp51 | 70.6% |  |  |  |
| Cyp51A | 80.95% | 100% |  |  | Cyp51A | 69.0% | 88.5% |  |  |
| Cyp51B | 90.48% | 90.48% | 100% |  | Cyp51B | 74.0% | 85.4% | 89.9% |  |
| Cyp51C | 66.67% | 52.38% | 57.14% | 100% | Cyp51C | 66.5% | 72.6% | 80.1% | 82.3% |
| AGXDTT | Cyp51 | Cyp51A | Cyp51B | Cyp51C | AGXDTT | Cyp51 | Cyp51A | Cyp51B | Cyp51C |
| Cyp51 | 100% |  |  |  | Cyp51 | 91.6% |  |  |  |
| Cyp51A | 83.34% | 100% |  |  | Cyp51A | 85.5% | 95.7% |  |  |
| Cyp51B | 83.34% | 100% | 100% |  | Cyp51B | 85.9% | 97.5% | 98.5% |  |
| Cyp51C | 50% | 50% | 50% | 100% | Cyp51C | 82.2% | 79.3% | 86.7% | 97.7% |
| EXXR | Cyp51 | Cyp51A | Cyp51B | Cyp51C | EXXR | Cyp51 | Cyp51A | Cyp51B | Cyp51C |
| Cyp51 | 100% |  |  |  | Cyp51 | 95.8% |  |  |  |
| Cyp51A | 100% | 100% |  |  | Cyp51A | 97.1% | 99.4% |  |  |
| Cyp51B | 100% | 100% | 100% |  | Cyp51B | 97.3% | 98.9% | 98.6% |  |
| Cyp51C | 100% | 100% | 100% | 100% | Cyp51C | 96.4% | 99.6% | 98.8% | 100% |
| SRS5 | Cyp51 | Cyp51A | Cyp51B | Cyp51C | SRS5 | Cyp51 | Cyp51A | Cyp51B | Cyp51C |
| Cyp51 | 100% |  |  |  | Cyp51 | 65.1% |  |  |  |
| Cyp51A | 70% | 100% |  |  | Cyp51A | 64.1% | 84.2% |  |  |
| Cyp51B | 70% | 70% | 100% |  | Cyp51B | 70.1% | 75.6% | 85.8% |  |
| Cyp51C | 80% | 90% | 80% | 100% | Cyp51C | 65.9% | 80.8% | 83.2% | 87.8% |
| PER | Cyp51 | Cyp51A | Cyp51B | Cyp51C | PER | Cyp51 | Cyp51A | Cyp51B | Cyp51C |
| Cyp51 | 100% |  |  |  | Cyp51 | 64.5% |  |  |  |
| Cyp51A | 100% | 100% |  |  | Cyp51A | 72.3% | 92.2% |  |  |
| Cyp51B | 100% | 100% | 100% |  | Cyp51B | 76.9% | 92.4% | 92.5% |  |
| Cyp51C | 100% | 100% | 100% | 100% | Cyp51C | 67.8% | 93.0% | 92.9% | 95.6% |
| FXXGXXXCXG | Cyp51 | Cyp51A | Cyp51B | Cyp51C | FXXGXXXCXG | Cyp51 | Cyp51A | Cyp51B | Cyp51C |
| Cyp51 | 100% |  |  |  | Cyp51 | 92% |  |  |  |
| Cyp51A | 100% | 100% |  |  | Cyp51A | 93.9% | 98.7% |  |  |
| Cyp51B | 100% | 100% | 100% |  | Cyp51B | 94.8% | 98.5% | 98.4% |  |
| Cyp51C | 90% | 90% | 90% | 100% | Cyp51C | 90.5% | 95.0% | 96.0% | 97.3% |
| SRS6 | Cyp51 | Cyp51A | Cyp51B | Cyp51C | SRS6 | Cyp51 | Cyp51A | Cyp51B | Cyp51C |
| Cyp51 | 100% |  |  |  | Cyp51 | 50.5% |  |  |  |
| Cyp51A | 33.34% | 100% |  |  | Cyp51A | 44.3% | 70.2% |  |  |
| Cyp51B | 33.34% | 77.78% | 100% |  | Cyp51B | 49.2% | 73.8% | 79.8% |  |
| Cyp51C | 44.44% | 77.78% | 77.78% | 100% | Cyp51C | 39.0% | 51.5% | 64.0% | 34.9% |

^1^Consensus sequences were generated by Geneious Prime.

^2^Similarity is based on Geneious Prime’s pairwise identity function.
