# Supplementary material for: Analysis of Cyp51 protein sequences shows 4 major Cyp51 gene family groups across fungi
Source: G3 (Bethesda). 2022 Sep 21;12(11):jkac249. doi: 10.1093/g3journal/jkac249 (PMC9635630; doi:10.1093/g3journal/jkac249)
Supplement: jkac249_Figure_S1 [file jkac249_figure_s1.pdf]

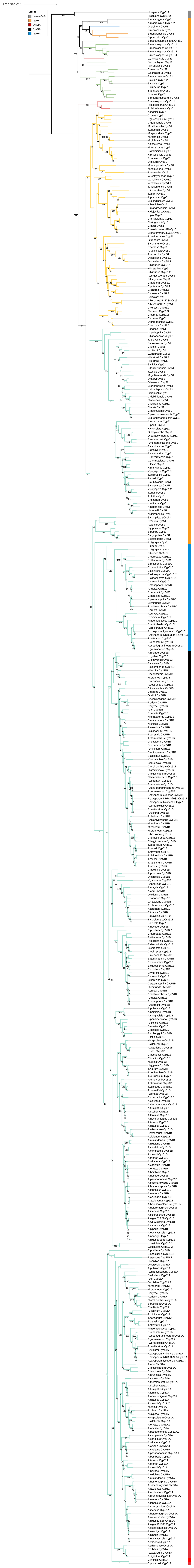
Tree scale: 1
Legend
Human Cyp51
Cyp51
Cyp51A
Cyp51B
Cyp51C
